# Interpretable EEG seizure prediction using a multiobjective evolutionary algorithm

**DOI:** 10.1038/s41598-022-08322-w

**Published:** 2022-03-15

**Authors:** Mauro Pinto, Tiago Coelho, Adriana Leal, Fábio Lopes, António Dourado, Pedro Martins, César Teixeira

**Affiliations:** 1 Department of Informatics Engineering, CISUC, Univ Coimbra, Coimbra, Portugal; 2 Epilepsy Center, Department Neurosurgery, Medical Center - University of Freiburg, Faculty of Medicine, University of Freiburg, Freiburg, Germany

**Keywords:** Epilepsy, Scientific data

## Abstract

Seizure prediction might be the solution to tackle the apparent unpredictability of seizures in patients with drug-resistant epilepsy, which comprise about a third of all patients with epilepsy. Designing seizure prediction models involves defining the pre-ictal period, a transition stage between inter-ictal brain activity and the seizure discharge. This period is typically a fixed interval, with some recent studies reporting the evaluation of different patient-specific pre-ictal intervals. Recently, researchers have aimed to determine the pre-ictal period, a transition stage between regular brain activity and a seizure. Authors have been using deep learning models given the ability of such models to automatically perform pre-processing, feature extraction, classification, and handling temporal and spatial dependencies. As these approaches create black-box models, clinicians may not have sufficient trust to use them in high-stake decisions. By considering these problems, we developed an evolutionary seizure prediction model that identifies the best set of features while automatically searching for the pre-ictal period and accounting for patient comfort. This methodology provides patient-specific interpretable insights, which might contribute to a better understanding of seizure generation processes and explain the algorithm’s decisions. We tested our methodology on 238 seizures and 3687 h of continuous data, recorded on scalp recordings from 93 patients with several types of focal and generalised epilepsies. We compared the results with a seizure surrogate predictor and obtained a performance above chance for 32% patients. We also compared our results with a control method based on the standard machine learning pipeline (pre-processing, feature extraction, classifier training, and post-processing), where the control marginally outperformed our approach by validating 35% of the patients. In total, 54 patients performed above chance for at least one method: our methodology or the control one. Of these 54 patients, 21 ($\approx$38%) were solely validated by our methodology, while 24 ($\approx$44%) were only validated by the control method. These findings may evidence the need for different methodologies concerning different patients.

## Introduction

Medication cannot effectively suppress seizures in patients with Drug-Resistant Epilepsy (DRE). These comprise a third of all patients with epilepsy. In addition to being harmful, often leading to severe injuries, seizures also bring social and psychological consequences, such as stress and stigma^1–4^. Additionally, less than 1% of patients with DRE are referred to a multidisciplinary epilepsy centre to confirm the diagnosis and evaluate whether they are candidates for epilepsy surgery^5^. An alternative to that treatment option could be the prediction of seizures to allow for an intervention, such as a warning device that timely warned patients to take rescue medication or avoid accidents^6–8^.

Epileptic seizure prediction has been studied for roughly 50 years using electroencephalographic (EEG) signal^9–11^. Researchers typically divide EEG signals into four periods: pre-ictal, preceding the seizure; ictal, corresponding to the seizure; post-ictal, a refractory period after the seizure; and inter-ictal, a seizure-free interval found between the post-ictal and the pre-ictal of consecutive seizures. It is essential to acknowledge that there is no clinical annotation of the pre-ictal period. Authors have not found a recurrent pattern among patients, but instead, they identified epileptic biomarkers specific for each patient^9,12–15^. Machine learning (ML) approaches are widely used in seizure prediction^4,10,12,13,16–18^. The standard ML study pipeline comprises modular and independent steps, including pre-processing, feature extraction, classifier training, and post-processing. Some studies also report the selection of a pre-ictal interval specific for each patient by performing a grid-search over different intervals (e.g. 10, 20, 30, 60, or even 240 min)^19,20^. Recently, researchers started considering deep learning (DL) as: (i) it has an increased potential to deal with temporal and spatial dependencies, (ii) it might extract non-linear relations between features, and (iii) it may also perform pre-processing and feature extraction automatically^15,21–27^. Nevertheless, there is a significant disadvantage: DL creates black-box models. Although some authors have contributed with significant efforts towards deep learning explainability^28,29^, we still might be far from delivering human-comprehensible explanations with these. As a consequence, clinicians may not be willing to trust such models in a high-risk decision scenario^30^. Developing a model that delivers human-comprehensible insights on its decisions may provide a higher degree of trust. Towards that end, using explainable models may be the solution to ensure that these models are stable, robust and possibly able to detect bias^29,31^. In other words, stressing the importance of explainability is a way to reinforce the highest priority of not compromising patient safety^32^, i.e., by not performing unnecessary interventions that may be harmful.

To the best of our knowledge, there are two main seizure detection/prediction systems clinically approved and tested in real life for a considerable period, registered in ClinicalTrials.gov, the largest database of clinical trials. Cook et al.^33^ presented a clinically accepted warning device, the Neurovista Advisory System. Sun and Morrel^34^ presented the Responsive Neurostimulation (RNS) system, which is the only commercially available device designed to deliver electrostimulation. Please note that the distinction between prediction and detection may be blurred as it may not be precise when to distinguish between them. Also, from the Neurovista Advisory System clinical trial, a prediction challenge was conducted at kaggle.com using patients who had the lowest seizure prediction performances^35^.

A standard prediction study attempts to simulate real-time by using long-term continuous recordings, where test data must be unknown by the model during training^9,14^. Typically, EEG data is acquired when the patient is under pre-surgical monitoring to assess surgery eligibility. In these conditions, patients suffer sleep deprivation and medication tampering, and therefore, these are not representative of daily life. Due to this, these studies can only be seen as proofs of concept^14,15^, since confounding factors such as circadian effects, medication alterations, stress, among others, induce alterations in brain dynamics which can modify the EEG signal. Also, from a classification point of view, a seizure is a rare event, which causes a significant class imbalance: the inter-ictal period is exceptionally long compared to the pre-ictal one^9^.

The study described herein follows our previous work^36^ where we proposed a seizure prediction model that provides highly interpretable decisions while searching for the optimal pre-ictal period. That approach was our first attempt to address the trust aspect when developing seizure prediction models. Additionally, the results from these models provided more information on the process of seizure generation. We can interpret our findings at lights of the bifurcation analysis^37,38^ while putting the pre-ictal state into a network perspective. In this analysis, when the dynamical system (brain activity) crosses a bifurcation, it can fall into the ictal state. The bifurcation concerns a point beyond which the inter-ictal state disappears, thus pushing brain activity inexorably towards the ictal state. This drift underlies the concept of measuring a pre-ictal tendency from EEG background analysis. This methodology also tackled the lack of synergy between all steps from the classical seizure prediction methodologies while handling time dependencies. To do this, we searched for discriminative features (in different and simultaneous time windows) for each patient that best predicted seizures with a logistic regression classifier by developing a personalised Evolutionary Algorithm (EA). EAs are based on a population of individuals (points in the search space) and are inspired by natural evolution. They are helpful for direct search, optimisation, and ML problems^39^. In sum, the individuals, defined by a set of features that best performed in seizure prediction, survived and proliferated. The remaining died and did not propagate their genes^39–41^. With this first study, we also demonstrated that we could obtain patient-specific knowledge by studying the phenotype from the EA populations. However, there were some limitations: (i) the analysis comprised of EEG data collected from a small group of 19 patients exclusively with temporal lobe epilepsy; (ii) we considered a small set of features from the literature were considered; and (iii) the phenotype study only accounted for the presence of genes and the discriminative power from correspondent logistic regression coefficients.

We present here a considerably improved version of our work that addresses the shortcomings mentioned above: (i) we tested a new methodology using data from 93 patients with several types of focal (temporal, frontal, occipital, and central) and generalised epilepsy; (ii) we added more features, still widely used in the literature in order to not lose interpretability potential; (iii) in our phenotype study, we also assessed gene interaction by using the a priori algorithm, a classical association rule method^42^. We now provide higher trust in our models’ performance while deepening current knowledge about seizure generation mechanisms. We also studied the aspect of patient comfort by assessing the electrodes that are providing discriminative information. We assumed that we provide more comfort to the patient by promoting solutions that do not require many electrodes (minimise the number of used electrodes) and focus on a particular brain region (minimise the number of analysed lobes). Towards that end, we replaced the single-objective EA reported in our first study^36^ into a multi-objective EA that searches for the best trade-off between seizure prediction performance and patient discomfort. Additionally, we created a control method, which is an adaptation from previous seizure prediction studies^17,33^, to better compare our obtained performance.

This paper presents a seizure prediction model developed using pre-surgical monitoring data that attempted to simulate real-life by testing chronologically and iteratively in unseen data (pseudoprospective approach). The envisioned real-life application concerns a patient-specific EEG scalp system up to a maximum of five electrodes to ensure patient comfort. Its pre-ictal period ranges from 30 to 75 min, with an intervention time of 10 min for each prediction, and provides the patient with sufficient time to avoid accidents and/or for rescue medication intake^43^.

## Materials and methods

**Figure 1:**
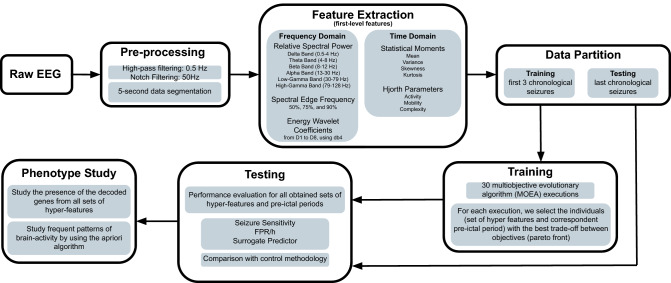
Flowchart of the proposed seizure prediction MOEA for each patient, comprising data processing, feature extraction, training, testing, performance evaluation and phenotype study.

Since we developed a patient-specific approach, we applied the following procedure to each patient: data pre-processing, feature extraction, training and testing, as depicted in Fig. 1. The raw EEG data of each patient was filtered and segmented into non-overlapping 5-second windows from which we extracted features. Next, we used the first three chronological seizures as input to the multi-objective EA (MOEA) and selected the individuals (set of five features and correspondent pre-ictal period) from the Pareto-front (made of three objectives) with the best trade-off between objectives (sample sensitivity, sample specificity, and patient comfort). Then, these five MOEA output features and correspondent pre-ictal periods were tested with the remaining seizures and compared with the seizure prediction method used as control. Since some parts of the MOEA are stochastic (initialisation and evolution operators), we can obtain, for each execution, a different set of Pareto-optimal solutions. Consequently, we repeated the MOEA execution 30 times. Then, a phenotype study was performed for all patients to understand the predictors’ decision mechanisms and infer about the possible patients’ seizure generation processes.

Concerning the MOEA output features, note that these were based on the concept of feature construction^44–46^. In our work, first-level features concern the ones directly extracted from the EEG. Then, we computed the second-level ones, which constitute the MOEA phenotype, by windowing and applying a mathematical operator to these features. Henceforth, we refer to first-level features as *features* and second-level features as *hyper-features*.

### Database

From the European Epilepsy Database, developed by the FP7 EPILEPSIAE project (www.epileisiae.eu)^3,6^, we selected 93 patients with DRE (53 males and 40 females, aged 35.60 ± 14.21 years) from the Universitätsklinikum Freiburg in Germany, from the Hôpital Pitié-Salpêtrière, Université Marie Curie, Paris, France, and from the Centro Hospitalar e Universitário, University of Coimbra, Portugal. In total, this dataset contains 518 seizures (279 for training and 238 for testing), 1116 h of training data and 3687 h of testing data ($\approx$ 5.12 months). For the training seizures, we only used the last recorded 4 h before each seizure to limit computational time. For testing, we used all available inter-ictal data without removing any segments. Our patient selection criteria were: (i) patients having a minimum of 4 recorded seizures separated by periods of at least 4.5 h; (ii) patients with EEG scalp recordings, placed according to the 10–20 system and using a sampling frequency of at least 256 Hz; (iii) patients with no more than one hour of EEG data missing for each seizure. All the analysed data were collected while patients were at the clinic under pre-surgical monitoring. The use of this data for research purposes has been approved by the Ethical Committee of the three hospitals involved in the development of the database (Ethik-Kommission der Albert-Ludwigs-Universität, Freiburg; Comité consultatif sur le traitement de l’information en matière de recherche dans le domaine de la santé, Pitié- Salpêtrière University Hospital; and Comité de Ética do Centro Hospitalar e Universitário de Coimbra). All methods were performed following the relevant guidelines and regulations. Informed written patient consent from all subjects and/or their legal guardian(s) was also obtained. Table S1 in Supplementary Material provides more information regarding the selected patients.

### Pre-processing and feature extraction

All patient data were downsampled to 256 Hz, segmented into 5-second non-overlapping windows, and filtered with: (i) a 50 Hz fourth-order notch filter (to remove the power-line interference) and (ii) a fourth-order Butterworth high-pass filter with a cut-off frequency at 0.5 Hz (to remove the DC component and minimise motion artefacts). For each time window in each electrode, we extracted 24 linear univariate features that were used in the literature^10,13,17,18,47,48^ and that are fast to compute. To understand each feature *a priori* expected added value, see Supplementary Material in the Feature Description section. It is also important to note that several features would be of interest^10,13,49–53^. We did not use them as they are not univariate (we would have to extract a considerably large number of features for each electrode) and are computationally heavier. In the time domain, we extracted the first four statistical moments (mean, variance, skewness and kurtosis) and the three Hjorth parameters (activity, mobility and complexity). As for the frequency domain, we extracted the relative spectral power of the delta (0.5–4 Hz), theta (4–8 Hz), alpha (8–12 Hz), beta (13–30 Hz), low-gamma (30–79 Hz), and high-gamma (79–128 Hz) bands, the spectral edge frequency at three different cut-off percentages (50%, 75%, and 90%) and the energy of each wavelet coefficient (D1 to A8, using the Daubechies 4 mother wavelet (db4)). As the frequency limit of gamma activity is not consensual among the scientific community, and its division into high-gamma and low-gamma is not uncommon^54^, we decided to divide it. Additionally, the gamma band powers may likely contain muscle artefacts as these recordings are extracranial. Therefore, we may not fully consider gamma-band powers as EEG features since these may represent physiological markers that predict seizures. Some authors^55^ report the difficulty of removing artefacts without eliminating good information; therefore, they may use raw signals.

### Multi-objective evolutionary algorithm

**Figure 2:**
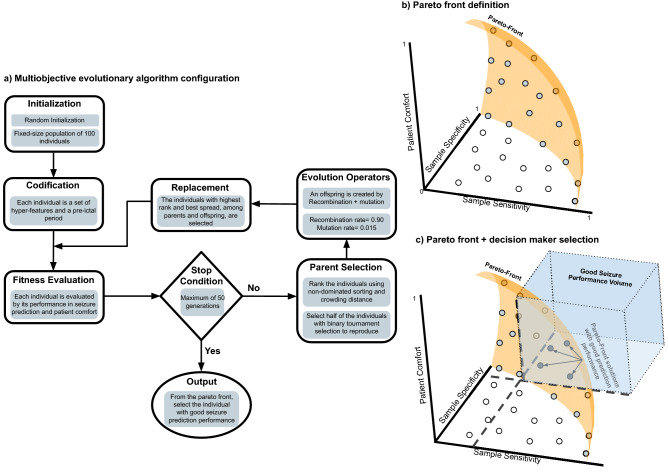
(**a**) The proposed MOEA configuration, (**b**) Pareto-front definition, and (**c**) the Decision Maker selection on the Pareto-front for selecting the individuals after the MOEA execution, where (**b**,**c**) reflect a problem formulated to simultaneously maximise all the objectives: two on seizure performance (sample sensitivity and sample specificity) and another on patient comfort.

Figure 2 depicts the employed MOEA, based on the Non-dominated Sorting Genetic Algorithm II (NSGA-II)^56^. Firstly, we randomly initialise our MOEA with a fixed-size population of 100 individuals. Each individual (set of five hyper-features and a pre-ictal period) is encoded by a chain of characters (genotype), which is further translated, from the problem context to the problem-solving space, leading to a possible solution (phenotype). Then, each individual is evaluated according to our fitness functions, which are two measures related to seizure prediction performance (sample sensitivity and sample specificity) and another representing patient comfort (based on the used electrodes and the used lobes). Based on the individuals’ fitness values and their spatial spread, two steps are performed: (i) ranking the individuals using non-dominated sorting along with the crowding distance^56^, and (ii) choosing half of the individuals (parents) to reproduce (variation operators) by using binary tournament selection (parent selection) until 100 offspring are generated. As variation operators, we used recombination with a rate of 0.90 (90% of times, two parents produced an offspring) and mutation with a rate of 0.015 (1.5% of offspring suffered a mutation). This mutation rate was based on the number of genes per individual. We then select the individuals with the best rank and higher spread among parents and offspring. These comprise the next generation of individuals. Evolution occurred over 50 generations. After the last generation, from the individuals with the best trade-off between the three fitness functions (Pareto-front or non-dominated individuals), we only keep the ones with a seizure prediction performance higher than a determined threshold (Decision Maker).

The Supplementary Material provides more information on the MOEA and a detailed explanation of the non-dominated sorting and crowding distance. Since a reduced computational time is needed to attain real-time applicability, we aimed for a fast convergence while achieving adequate solution diversity. Thus, that was the reason for opting for NSGA-II. Although this strategy may not usually produce an optimal solution, we believe that it approximates the global optimum within a reasonable amount of time^56,57^. Each execution lasted approximately 2 h on a machine equipped with an Intel Core i5-3230M 2.6 GHz processor, 8 GB of RAM, running on macOS Mojave 10.14.6 and using Python 3.7 on Spyder 4.0.1.

#### Encoding and variation operators

**Figure 3:**
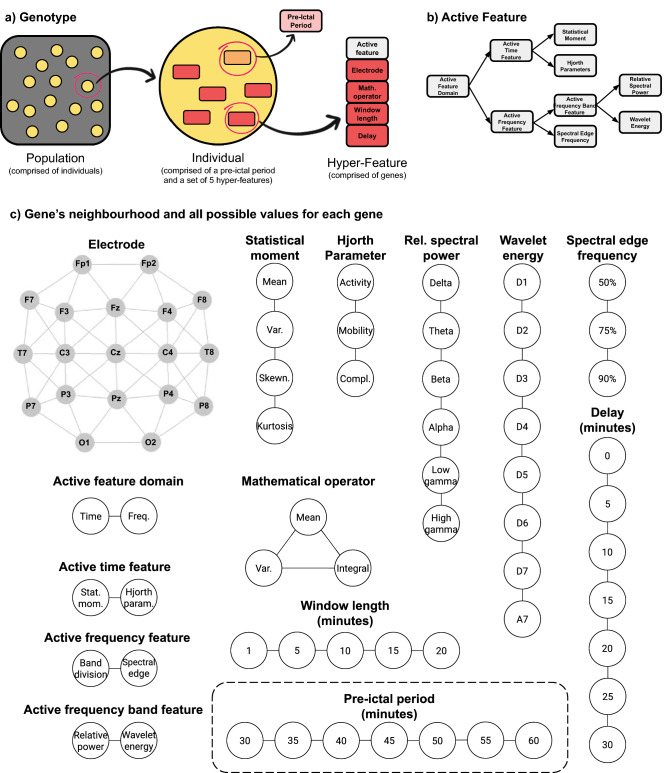
(**a**) Illustrated scheme of genotype. A population is comprised of a group of individuals, where each one is constituted by five hyper-features. Each hyper-feature is encoded with thirteen genes: nine for the first-level feature, as well as delay, electrode, mathematical operator, and window length. (**b**) Active feature decoding, or in other words, how to decode the first-level feature from the genotype. (**c**) Gene’s neighbourhood and all possible values for each gene, which allows the application of variation operators.

Figure 3 depicts the idea behind genotype, phenotype decoding, and mutation. A population is comprised of a group of individuals, where each is constituted by five hyper-features. Each hyper-feature is encoded with 13 genes: nine for the first-level feature (active feature group, active time feature, active frequency feature, active frequency band feature, statistical moment, Hjorth parameter, relative spectral power, wavelet coefficient energy, and spectral edge frequency), as well as a delay (minutes of a given feature before the pre-ictal period), electrode, mathematical operator, and window length (as depicted in Fig. 3a).

The mapping from genotype to phenotype consists of the same rationale as in Pinto et al.^36^: (i) finding the first-level feature that will be decoded to the phenotype for each hyper-feature, using the mentioned nine genes (as shown in Fig. 3b); (ii) constructing each hyper-feature by windowing the decoded first-level feature from the given electrode within the window length gene and, afterwards, by applying the respective mathematical operator; and iii) placing each hyper-feature chronologically in a timeline using the delay gene, according to the pre-ictal period. This allows us to analyse a sequence of events with a given interval instead of the traditional analysis of feature alterations in that same interval. Since the pre-ictal period is now included in the genotype, the MOEA also automatically searches for the optimal one. After constructing the five hyper-features and placing them chronologically, it is possible to evaluate a phenotype using the fitness function by performing a typical seizure prediction pipeline (sliding window analysis, classification, and regularisation).

Figure 3c shows all possible values for each gene, along with its neighbourhood. The latter is necessary to apply the variation operators, recombination, and mutation. The neighbourhoods were designed by considering the relationship between gene values (see Supplementary Material for more details on neighbourhood definition). The variation operators follow the same rationale as the ones in Pinto et al.^36^. The mutation operator concerns a unitary step that causes a random and unbiased change. Recombination is a stochastic operator that combines the genetic information from two parents (individuals) into one or more offspring^39^. It performs the recombination of all paired hyper-features (by order of similarity) and the recombination of the two pre-ictal periods.

#### Fitness functions and decision maker

**Figure 4:**
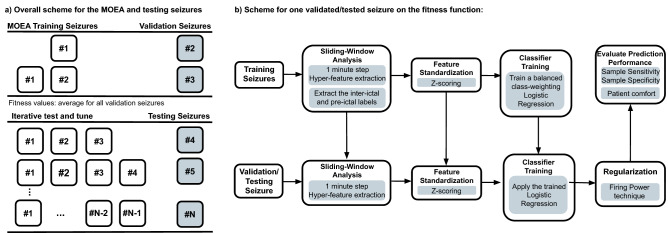
An illustrated scheme of the iterative retraining and validation in the MOEA input seizures. With the selected hyper-features, an iterative test and tune procedure is made (**a**). Scheme for one tested seizure on the fitness function (**b**).

Figure 4 depicts the evaluation of each individual, which is performed iteratively (retraining the logistic regression classifier with new seizures) using the metrics typically used in seizure prediction. The seizures evaluated by the MOEA are referred to as validation seizures. After the MOEA, the same procedure is used to test new seizures, referred to as testing seizures (Fig. 4a). Thus, for each validation/testing seizure, we extract hyper-features and labels from the previous seizures through sliding-window analysis using a 1-min step. Lastly, we standardise the feature set with *z*-scoring.

We used the standardised features to train a classifier that balances samples according to a class weight which is inversely proportional to its frequency of occurrence. We opted for a logistic regression as it is an intrinsically interpretable model with low computational requirements^29^. Furthermore, it is computationally light. Next, we apply a similar process to the validation/testing seizure using all the parameters (mean and standard deviation for *z*-scoring) and models obtained from training. Then, we apply the Firing Power^58^ regularisation technique to smooth the classifier output and to make it more robust to noise. The latter works as a moving-average filter such that when a given threshold is exceeded, it triggers an alarm. We decided not to include the Firing Power threshold in the MOEA genotype as it would be another parameter to tune and increase computational time. Therefore, we defined a reasonable value of 0.7^36^ without any tuning.

We evaluated the seizure prediction models using three metrics. Two of them are based on seizure prediction performance: sample sensitivity $S_{ss}$ (ratio of samples classified as pre-ictal within all pre-ictal samples) and sample specificity $S_{sp}$ (ratio of samples classified as inter-ictal within all inter-ictal samples). The third metric, *Pcf*, is related to patient comfort and concerns the number of different electrodes and their position on the scalp concerning lobes. We assumed that we provide more comfort to the patient (*Pcf*) by promoting solutions that do not require a large number of electrodes ($N_{electrodes}$) and that focus on a particular region of the brain (minimise the number of analysed lobes $N_{lobes}$). This last metric was computed according to Eq (1), where the term 1.25 is just a normalisation factor so that its range is [0, 1]:

1 $$\begin{aligned} Pcf=1.25\left( 1-\frac{N_{electrodes} \times N_{Lobes}}{N_{electrodes} \times 5_{features}}\right) . \end{aligned}$$

This way, we obtain a $Pcf=1$ when only one electrode is used ($N_{electrodes}=1$ and $N_{Lobes}=1$). When five electrodes from different five lobes are used ($N_{electrodes}=5$ and $N_{Lobes}=5$), we get a $Pcf=0$. We try to minimise the number of electrodes and the number of used lobes to maximise comfort. It is worth noting that there were other possibilities to handle spatial closeness, such as computing the total distance of each electrode to the remaining ones in the form of a graph. Nevertheless, we wanted to understand a possible relation concerning brain lobes.

After each MOEA execution has been completed, we implemented a Decision Maker (DM) to select which individuals from the set of Pareto-optimal solutions will be used in the testing phase. The DM selects individuals with a sufficiently high fitness score for the seizure prediction objectives (sample sensitivity and specificity) to use for testing. This restriction was implemented because even though the MOEA finds solutions corresponding to the analysis of a low number of electrodes/lobes, some of these Pareto-optimal solutions may present inadequate classification performance within the training set. Thus, we set a minimum fitness threshold of 0.9 for $S_{ss}$ and $S_{sp}$ metrics. When no solutions could be selected within a run, the threshold was decreased further to 0.8 to guarantee the selection of at least one individual. We did not define any minimum threshold for patient comfort.

#### Training, testing, statistical validation, and performance comparison

We would only run the MOEA once in a real-life application and select one individual from the Pareto-optimal set to predict new seizures. We are still in a research stage, so we need to understand how stochasticity affects performance. Thus, we executed each MOEA 30 times for each patient and then tested the obtained hyper-features on unseen data. The testing phase was performed using the same pipeline for the fitness function (see Fig. 4b) where we also included a refractory period that follows an alarm with the duration of the pre-ictal time. These periods were excluded from the False Positive Rate per Hour (FPR/h) metrics so that we consider only the period during which false alarms can be triggered^9^.

We measured our models’ performance with seizure sensitivity $S_{s}$ (ratio of correctly predicted seizures), with FPR/h (ratio of false alarms raised until seizure onset excluding the refractory period). Then, we performed a Surrogate analysis^36,59^ to understand if the methodology is performing above chance level. We also computed these metrics for a control method inspired by standard machine learning prediction approaches, particularly in the work of Cook et al.^33^. Although the methods were built based on data from intracranial electrodes, it is the most relevant clinical trial that used an ML approach on a warning device. We refer to Supplementary Material for more details on the control method.

The obtained patient models were statistically validated as follows: a method performs above chance level when its seizure sensitivity is higher than the surrogate one, with statistical significance of $\alpha =0.01$ using a one-tailed *t-test*. We also verified if the set of validated patients had statistical significance in the same way as Alvarado Rojas et al.^4^. Considering a statistical significance of $\alpha =0.05$, the probability of observing, for at least *i* of *I* (patient-models) executions that outperformed the surrogate predictor, is given by:

2 $$\begin{aligned} P_{binom}(i,I,\alpha ) = \sum _{j=i}^{I}\left( {\begin{array}{c}I\\ j\end{array}}\right) \alpha ^{j}(1-\alpha )^{(I-j)}. \end{aligned}$$

#### Phenotype study

We performed a phenotype study to analyse the overall independent influence of each gene value on the population^36^. Thus, we assigned a binary value (0 or 1) for each gene value that corresponds to its presence in a hyper-feature. We computed the presence binary value for all hyper-features from selected Pareto-optimal individuals. However, these metrics cannot provide information on interaction, i.e., information regarding which features always appear in the presence of others. Therefore, we also implemented the *apriori* algorithm^42^ that aims at finding frequent patterns in the obtained phenotypes (association learning). By using association rules, we tried to find subsets of gene values that frequently appear together, and therefore, have a high probability of describing seizure generation processes.

## Results

In one subsection, we present the results for the MOEA and control method and, in another subsection, the phenotype study.

### MOEA and control method performance

**Figure 5:**
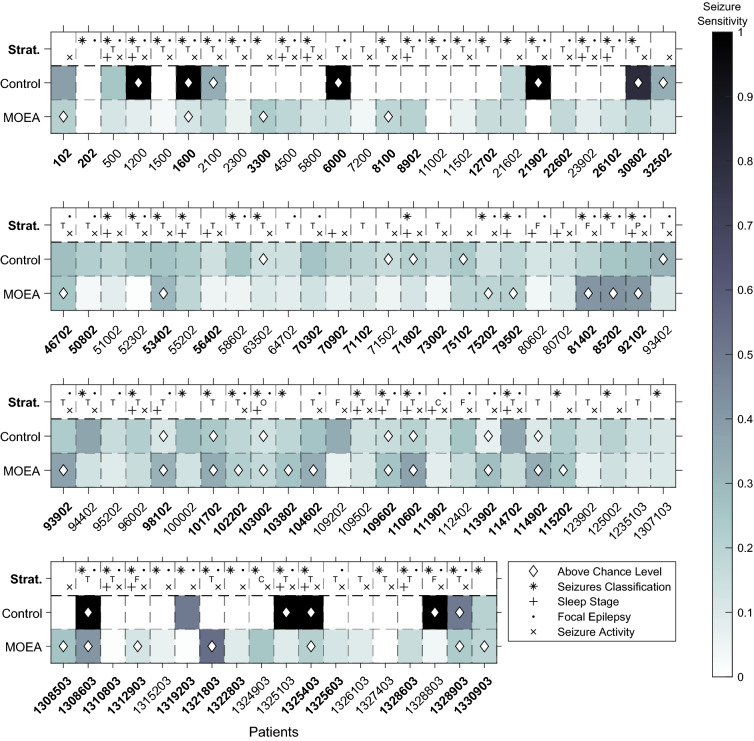
The test performance of all patient models, for the MOEA and the control method. Colour represents the seizure sensitivity, while the diamond shape means that the models performed above chance level. On the top line (Strat.), patient stratification is presented concerning seizure classification (*, FOA or FOIA/focal to bilateral tonic-clonic seizures), sleep stage at seizure onset (+, awake/sleep), type of epilepsy (., focal/generalised) and annotated activity pattern (x, rhythmic/non-rhythmic), and seizure focus that is lobe-specific (temporal (T), frontal (F), central (C), parietal (P), occipital (O)).

Figure 5 depicts the results for the testing seizures, both for the MOEA and the control method, along with patient stratification. Colour represents seizure sensitivity (0-1), while the diamond-shaped marker represents the patient models that outperformed the surrogate predictor, or in other words, performed above chance. For 30 patient models (32%), we observed a performance above chance using the MOEA, whereas the control method validated 33 patients (35%). By inspecting Fig. 5 and the test results in full detail (Table S2 in Supplementary Material), we can see that the MOEA obtained lower sensitivities and lower FPR/h values when compared to the control method. Although the MOEA obtained six models that were statistically validated while presenting an adequate FPR/h (< 0.15^60^), we believe that high sensitivity is missing for claiming its use in real-life. However, the control method presented models for eight patients (202, 3300, 6000, 8902, 21902, 26102, 1310803, 1322803) with maximum sensitivity and adequate FPR/h. Nevertheless, it is worth noting that seven of these patients only had one seizure for testing, while patient 8902 had two. It is important to mention that our methods might be overfitted (overestimated) to the training seizures, as our training results are considerably higher than testing. While the average fitness in the validation seizures (Table S2 from Supplementary Material) was 0.97 ± 0.02 for sample sensitivity and 0.96 ± 0.02 for sample specificity, for testing, we obtained an average seizure sensitivity of 0.16 ± 0.11 and an average FPR/h of 0.21 ± 0.08. Concerning the patient comfort (Table S2 from Supplementary Material), it is worth noting that the ideal scenario of having only one electrode was not achieved in any patient model. In fact, the patient comfort objective ranged from 0.50 ± 0.19 (patient 111902) to 0.86 ± 0.11 (patient 10962). Thus, in general, the MOEA opted for three to four electrodes to capture pre-seizure brain dynamics.

Table 1 shows test results for the set of patients and patient stratification. We based our stratification on seizure classification (focal onset aware seizures (FOA) or focal onset impaired awareness seizures (FOIA) only), seizure activity (only rhythmic), vigilance state at seizure onset (awake only), seizure focus in one hemisphere/more than one hemisphere (focal/generalised epilepsy), temporal lobe epilepsy, seizure focus not restricted to a single lobe, and concerning surgery decision (localised/not localised). Due to the small number of patients, we did not consider other stratified groups. For example, there were only seven patients available with frontal lobe epilepsy. In the stratification regarding seizure classification, activity, and vigilance state at seizure onset, a patient was selected if a given criterion was met both in the training and testing seizures. Seizure classification and the patients with a seizure focus in non-specific lobes were the only criteria that considerably improved the percentage of validated patient models (39% and 38%, respectively) for the MOEA. Moreover, in the FOA or FOIA seizures stratified groups, the ratio of validated patient models was higher on the MOEA methodology than on the control.

**Table 1:** Test results for the overall set of patients (sensitivity, FPR/h, and ratio of patients performing above chance, both for the MOEA and the control method), and for stratified sets of patients.

Stratification	Number of Patients	MOEA			Control		
		Sensitivity	FPR/h	Patients performing above chance (0–1)	Sensitivity	FPR/h	Patients performing above chance (0–1)
Focal epilepsy	55	0.15 ± 0.12	0.21 ± 0.08	0.33	0.31 ± 0.39	0.67 ± 0.92	0.35
Generalised epilepsy	33	0.17 ± 0.11	0.21 ± 0.09	0.33	0.35 ± 0.37	2.18 ± 7.13	0.36
Temporal lobe epilepsy	61	0.16 ± 0.12	0.22 ± 0.09	0.30	0.32 ± 0.40	1.54 ± 5.30	0.33
**Non-specific lobe**	**21**	**0.15** ± **0.08**	**0.21** ± **0.06**	**0.38**	**0.38** ± **0.37**	**1.01** ± **2.31**	**0.48**
**Only FOA or FOIA seizures**	**59**	**0.17** ± **0.12**	**0.22** ± **0.07**	**0.39**	**0.28** ± **0.38**	**0.82** ± **1.57**	**0.31**
Localised focus	49	0.15 ± 0.10	0.22 ± 0.08	0.31	0.28 ± 0.36	0.66 ± 0.91	0.29
**No localised focus**	**44**	**0.15** ± **0.12**	**0.20** ± **0.08**	**0.30**	**0.37** ± **0.39**	**2.18** ± **6.33**	**0.45**
Awake-only onset seizures	29	0.16 ± 0.09	0.21 ± 0.07	0.31	0.33 ± 0.38	1.05 ± 2.04	0.38
Rhythmic activity only	59	0.15 ± 0.11	0.22 ± 0.09	0.29	0.32 ± 0.36	1.54 ± 5.45	0.37
**Overall**	**93**	**0.16** ± **0.11**	**0.21** ± **0.08**	**0.32**	**0.32** ± **0.38**	**1.31** ± **4.43**	**0.35**

Stratification concerned patients the following criteria: (i) focal epilepsy patients (seizure focus in only one hemisphere), (ii) generalised epilepsy patients (seizure focus in more than on hemisphere), (iii) temporal lobe epilepsy patients (seizure focus only in temporal lobe), (iv) patients whose seizure focus is not lobe-specific, (v) patients suffering only from FOA/FOIA seizures, (vi) patients whose surgery was offered (localised focus), (vii) patients whose surgery was not offered (not localised focus), (viii) patients that only experienced seizures while they were awake., and (ix) patients with pre-seizure activity annotated as rhythmic by clinicians.

The overall results and the best performing stratified groups are in [bold].

### Phenotype study

**Figure 6:**
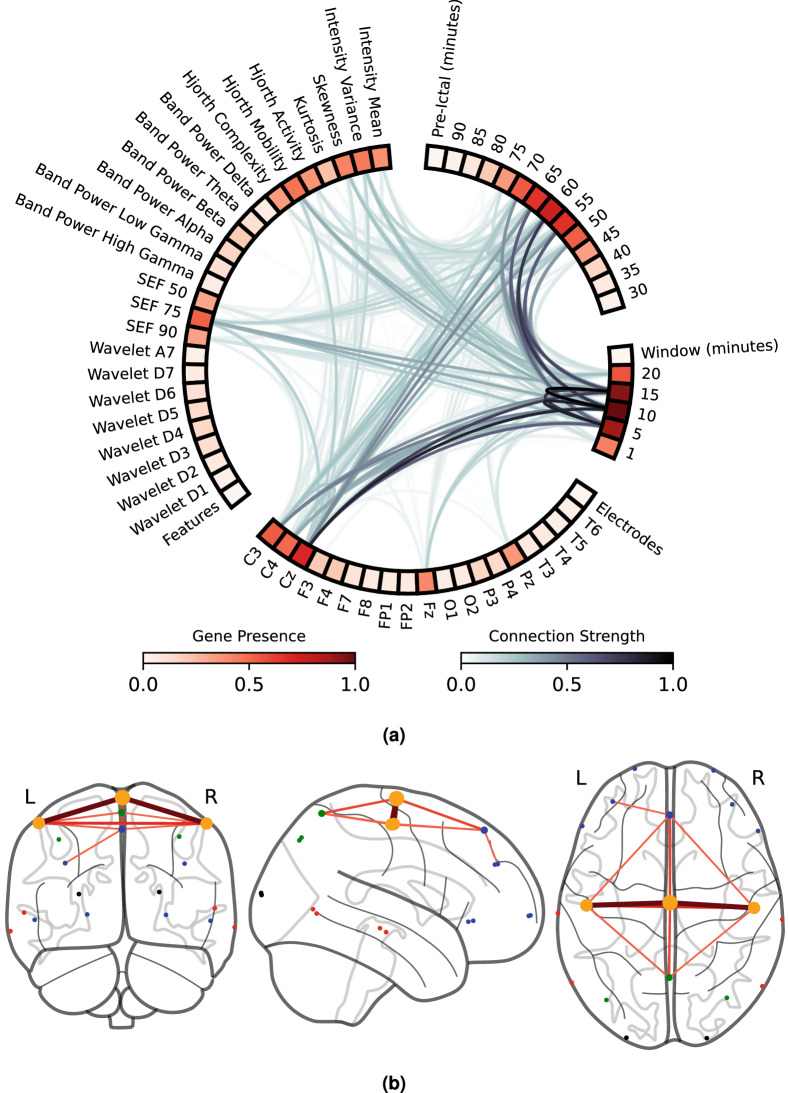
Gene interaction study for all patients. (**a**) Gene presence (red colourmap) and gene interaction (grey colourmap) in a chord diagram plotted using MNE Python library^61^. Gene presence is the ratio of times a given gene is present. Gene interaction is given by summing the association lift measures calculated with the a priori algorithm and then normalised to a 0–1 scale. (**b**) Brain connectivity was plotted using Nilearn Python library, and the 10–20 system electrodes were converted to the MNI coordinates^62^. Node size corresponds to the electrode presence, while edge colour and thickness provide connection strength (association lift). Node colour represents brain lobes (blue: frontal, orange: central, green: parietal, black: occipital, and red: temporal). A priori algorithm parameters: minimum support of 0.07, minimum confidence of 0.10, and minimum lift of 1.00.

We analysed the patients ’ phenotype to demonstrate the MOEA potential to unravel pre-seizure knowledge. Here we present a phenotype study for all patients, which explores gene importance similarly to our previous study (refer to Figures S.1 and S.2 in Supplementary Material). The results indicate that most solutions were obtained from three electrodes across two lobes and localised in two regions (we considered three regions: left and right hemispheres and the central part). Additionally, we improved the phenotype study by providing an additional gene interaction study by showing which gene values appear along with other gene values and how to present them intuitively. We also performed an additional analysis (see Figure S.3 from Supplementary Material): we inspected the most common solutions within each patient, particularly the obtained pre-ictal period and the number of different electrodes, lobes, regions, window lengths, and delays. With this analysis, we can confirm that most patients had a set of three electrodes, two lobes, and two regions as the most common solution. Moreover, no patient had the same window length for all features and used at least three delays. The most common pre-ictal periods were 50 and 55 min, despite some presenting a 60- or a 45-min one. Figure 6a represents the gene presence (red colormap) and gene interaction (gray colormap), and (b) represents brain connectivity. We calculated gene interaction using the a priori algorithm^42^: first, we found frequent associations between gene values, and then we used the association lift measure^42^ measure to map interaction strength. We searched for lift ratios larger than 1.00, which means that the two association items are more likely to appear together than separated. The larger a lift ratio is, the more significant is a given association rule. Brain connectivity was computed based on association rules between electrodes and using association lifts higher than 1.0.

The most important features were Spectral Edge Frequency (SEF) 75, Hjorth Mobility/Variance, and skewness. These features have the highest gene presence among all features and present the highest number of strong interactions. The same gene interaction study was also conducted for the electrodes (with a prevalence of C3, C4, CZ, PZ, and Fz) and pre-ictal periods (prevalence of 45 to 75-min intervals). Concerning window length, all windows were present in phenotype, with a higher frequency observed for the 10- and 15-min windows. We can also find gene interactions between feature-to-feature, electrode-to-electrode, and window-to-window genes. We highlight that the most robust relations were observed for the window-to-window gene interactions. These findings demonstrate the importance of simultaneously analysing different time windows. We can obtain a similar conclusion by inspecting Figure S2 in Supplementary Material, where the MOEA chose different delays about 99% of times and 99% of times concerning different window lengths. We also observed electrode-to-electrode interactions, which we interpret here as a manifestation of brain connectivity. Some connections arise across the central, frontal, and parietal lobes. The whole-brain state’s importance demonstrates the difficulty of finding a good set of predictive features while providing maximum patient comfort (minimising the number of electrodes and restricting electrode placement region). In our case, in addition to having patient comfort as an objective of the MOEA, we considered it pertinent to inspect brain activity across different lobes and hemispheres.

## Discussion

We present the discussion in several subsections: (i) the MOEA performance along with a comparison with the control method, (ii) comparison with other studies, (iii) the added value of this work, and (iv) study limitations.

### MOEA performance and control method

Despite the control method being marginally better than our proposed MOEA in terms of validated patients, we believe the MOEA has potential and brings added value. It is interesting to verify that only nine patients had both methodologies (MOEA and control) performing above chance. In total, it was possible to find statistically validated models for 54 patients (58%). Of these 54 patients, 21 ($\approx$38) were only validated by the MOEA. We believe these results evidence that we may need to vary the selected features and classification models and use significantly different methodologies in a complementary way to capture patient-specific pre-seizure patterns. Moreover, from the 33 validated patients by the control method, six (1328603, 1325603, 1308503, 109602, 98102, 71802) presented an FPR/h > 1.50, and ten patients presented an FPR/h > 1.00 (1328903, 1328603, 1325603, 1308503, 109602, 98102, 73002, 71802, 71102, 50802). In our MOEA, no patient presented a mean FPR/h higher than 0.37, although the sensitivity values were significantly lower. Lastly, the obtained model from the control methodology is a random forest with several features, ranging from 3 to 20 features. In fact, the average number of used features was 9.25 ± 6.26. Our MOEA only uses five features for computational reasons, limiting the number of electrodes to five.

Although the MOEA evaluation metrics are different from the testing ones, high sample sensitivity and high sample specificity lead to high seizure sensitivity and low FPR/h. This evidence can be explained due to confounding factors, as our database concerns patients under pre-surgical monitoring submitted to medication withdrawal to study the epileptic focus. We found an interesting relation between the number of tested seizures and validated patient models when exploring our results. While we found a negative correlation coefficient ($\rho =-0.15$) when analysing the control method, we obtained an almost null coefficient ($\rho =-0.01$) for the MOEA. These results suggest that retraining the logistic regression after a new seizure occurs might improve seizure performance. By doing this, we are intrinsically assuming that the selected features by the MOEA can deal with new concept drifts, and that is why we constructed our fitness function based on iterative training. We believe these results would be more significant if we would iteratively run the MOEA to update the set of selected features instead of just training the logistic regression with upcoming tested seizures. By re-selecting new features, we would better handle concept drifts. We decided not to do it due to computational costs.

Nevertheless, performing MOEA update for each new upcoming seizure can be considered for real-time applications, as running one execution of the MOEA takes approximately 2 h, more than twice shorter than the analysed inter-seizure independence interval (4 h 30 min). We only tested this methodology once and did not try to improve results. We also decided to keep a 10-min intervention time^60^ to provide the patient sufficient time to prepare for a seizure. This period is also compatible with the intake of diazepam rectal gel (the only FDA drug approved for seizure cluster and which might be tested for prevention) that needs 5–10 min to take effect^43^.

### Other studies comparison

There are some studies presenting seizure prediction models developed that use EPILEPSIAE^4,12,18,47^. Some works^12,18,47^ cannot be directly compared to our methodology, as they developed several models and presented the best ones based on test results, which does correspond to real-life performance. The study from Alvarado Rojas et al.^4^ is comparable with our methodology due to the following: (i) model selection was not based on testing performance but on training data only; (ii) it contains 53 patients (in total 531 days and 558 clinical seizures); and (iii) they used a threshold classifier, which is intuitive, despite the use of features that may be difficult to understand from a clinical point of view. Their results outperformed ours in seizure sensitivity ($\approx 0.66$), but the MOEA was better in terms of FPR/h ($\approx 0.33$) and performance above chance level for a higher ratio of patients ($7/53\approx 0.13$). We also consider our methodology more intuitive: each model uses a binary decision based on a threshold (logistic regression) and is comprised of a set of five widely-used and intuitive features. Direito et al.^17^ also allows a good comparison, being the study reporting the analysis of the largest group of patients from EPILEPSIAE (218 patients). Their work also outperformed ours in sensitivity (0.39), but statistical validation was achieved for about 11% of the patients. Nevertheless, it is worth noting that they used a random predictor^63^ to perform statistical validation instead of the surrogate analysis.

We emphasise that our performance is still far from being ideal for a real-time application, despite the ratio of validated patient-specific prediction models remaining similar to our previous study^64^. It is worth noting that three patients (16202, 60002, 98202) of the previous work were discarded in this study, as the correspondent recordings presented significant gaps of missing data (over one hour). In terms of sensitivity, the results were also not satisfactory. Concerning FPR/h, the new results were better. Due to this, we believe our models do not fully capture the complexity of the seizure generation processes. Results also changed within the same patient when comparing both works. Patient 55202 is one example: it presented significant EA results, but not with the MOEA. We used the first 60% of seizures in the first EA to train the algorithm, and we tested the last 40%. In the MOEA, since we wanted to have more testing seizures, we used the first three seizures to train the algorithm and tested the remaining ones. Patient 55202 has eight seizures. Therefore, the previous EA was trained with five seizures and tested with three seizures, while the MOEA was trained with three seizures and tested with five. Performance may increase from having more seizures as training data.

The use of deep learning may theoretically yield improved results by enabling more complex and hence less intrinsically interpretable models, however, at the cost of losing clinical interpretation^15^. Furthermore, our study comparisons with our study are limited by choice of the statistical validation method. All of the studies mentioned above use the random predictor^63^ while we used a surrogate predictor. The latter is flexible, and it adapts better to the data. Moreover, the choice of the surrogate predictor was bounded by the considerable number of models obtained for each patient and the small number of tested seizures per patient. Having a small number of seizures to test the models can significantly discourage the use of a random predictor^9,59^. We believe the surrogate predictor provided a more solid validation to our work for these reasons.

As previously mentioned, we put efforts into building models to be applied in a real-time scenario, where we used 10-min intervention time and extracranial recordings. We focused on scalp EEG as we wanted to understand the importance of the whole brain state and not only the seizure onset zone. This might explain that this study of many different types of epilepsy has the same outcome as the earlier study of temporal lobe epilepsy only. Like in the previous EA, we stress that iteratively re-selecting our features, by executing the MOEA periodically, would even consider that the epileptic network would imply considering a dynamic epileptic network, rather than a static one, which may produce more insights into the brain dynamics^14^.

### Added value

With this work, We aim to comply with current legislation, specifically with the 2018 General Data Protection Regulation (GDPR) for European citizens and the European economic space. The GPDR’s article 22 presents the first steps towards legislation on algorithm explainability for high-risk decisions based on personal data. It provides patients with the right to have an explanation for any algorithm decision and also gives them the right to question those decisions. One of the main advantages of our seizure prediction model is to explore the obtained phenotype to find patients’ pre-ictal patterns.

The inclusion of our patient comfort metric has allowed us to obtain more comfort EEG configurations for most patients theoretically. Since we obtained a set of three electrodes for 68 patients (73%) and a set that uses two lobes for 86 patients (92%), we can consider that patient comfort was relevant within our MOEA. Without this metric, we assume that we would get an ideal solution of 4/5 electrodes widespread across several lobes. However, we are aware that the optimal way to conclude the comfort relevance would be the following: (i) execute the MOEA for all patients without the comfort objective, (ii) by performing the phenotype same study on the number of the electrodes and lobes, and (iii) by comparing the obtained output. It is worth noting that not all MOEA solutions present the same set of electrodes within each patient. Thus, one can choose one configuration that was not the most common within the obtained solutions as long as it provides more comfort and maintains its performance levels. To better understand the relevance of the number of chosen electrodes and lobes, one can visualise a histogram for all solutions, for all patients, in this paper GitHub page. We also present five examples (patients 1200, 12702, 55202, 81402, and 1319203) in Supplementary Material in Electrodes and Lobes Discussion section. An overall study can also be seen in Figure S.3 from Supplementary Material, developed using the mode operator (most frequent value) for each patient. We chose these five patients as these cases represent all found case scenarios concerning the electrode/lobe topic. Additionally, when analysing the number of occupied regions (left hemisphere, central part, right hemisphere), the majority of occupied regions is two. This is a limitation that we must address in the future by including a factor that minimises the number of occupied regions in our patient comfort objective.

We analysed the impact of patient comfort on performance. For each patient, we made two scatter plots to access the patient comfort impact: (i) scatterplot(comfort, sensitivity) and (ii) scatterplot(comfort, FPR/h). We also computed the Pearson correlation coefficient between comfort and sensitivity and between comfort and FPR/h to assess this impact. We obtained null correlation values for sensitivity and FPR/h when analysing the overall correlation between all patients. However, by analysing each patient separately, we were able to find several case scenarios. For 33% of patients, overall performance increased with more comfortable electrode configurations. For 32% of patients, overall performance was maintained, and for 35% of patients, overall performance decreased with more comfortable configurations. We considered a sensitivity increase/decrease with comfort when the correlation between sensitivity and comfort was higher/lower than 0.10/ − 0.10. When the absolute correlation value was lower than 0.10, we considered that performance was maintained despite the used electrode configuration. We applied a similar rationale to FPR/h: we considered a sensitivity increase/decrease with comfort when the correlation between sensitivity and comfort was higher/lower than − 0.10/0.10.

A decrease in performance was expected, and maintenance could be expected as well. Nevertheless, an increase in performance was not expected. This increase could be due to the overfitting of configurations using more electrodes. By using fewer electrodes, model generalisation may be more easily achieved. Thus, we may conclude that, for some patients, the most comfortable configurations may be used as performance is not negatively affected. The analysis for each patient separately can be found in our Github in the folder Impact of Comfort in Performance. In our Supplementary Material, one can also see some examples (patients 1500, 58602, and 21602).

### Limitations

Due to computational speed, we only used the last 4 h before each training seizure. We believe this is the most significant limitation of our methodology, as it is strongly advised^9^ to use all available data from training seizures. Using all available data would increase inter-ictal data representativity and identify and deal with reoccurring confounding variables. Also, the remaining parameters, such as the 1-min step and number of used features, were reasonably chosen based on the computation time, without any tuning on the test results. Even though our results may be low in terms of sensitivity and FPR/h, we worked with pre-surgical monitoring long-term data without pre-processing, except filtering.

It is also essential to discuss the obtained pre-ictal periods. All patients presented a similar pre-ictal period of around 50 min. We analysed all solutions’ pre-ictal periods from all patients. We present in Supplementary Material some examples for patients 21602, 21902, 30802, and 32502, which had, as mean pre-ictal period, the following values, respectively: 49.91 ± 9.55, 51.40 ± 9.55, 51.73 ± 8.31, and 53.14 ± 8.70 min. We also present the most frequent pre-ictal period (the mode) for each patient. The MOEA can find solutions for many possible pre-ictal periods in all patients, but when we analyse the most frequent one (the mode) for each patient, it can range from 35 to 60 min (see Figure S.3 from Supplementary Material). An overall can also be seen in Figure S.3 from Supplementary Material, developed using the mode operator (most frequent value) for each patient.

It may be desirable to have a clear pre-ictal period for each patient. However, it may also be helpful to choose an MOEA solution based on the patient preferences concerning a trade-off between performance and the pre-ictal period. Longer pre-ictal periods may bring better performances but may also cause considerable stress. We believe these findings are a built-in bias. Our best hypothesis concerns that the pre-ictal period changes the number of samples belonging to each class (pre-ictal, inter-ictal) in problems with high-class imbalance (as is the case of seizure prediction), sensitivity and specificity might be significantly affected. As sensitivity and specificity are metrics used in our MOEA, it is very likely the existence of a built-in bias during the search procedure of the algorithm that drives the pre-ictal period towards values that optimise different trade-offs between sensitivity and specificity through class balancing.

Also, we have 31 patients with only one seizure for testing. We tried to make a good compromise between data quality and the number of patients, which resulted in 93 patients. It is difficult to perform a seizure prediction study, particularly patient-specific, with such a high number of patients due to data constraints. If we raised the minimum number of seizures to five, we would lose 31 patients, representing a third of all used patients. On average, we tested 2.57 seizures per patient.

Lastly, using five electrodes may result in a severe undersampling of the underlying cortex in several patients. However, this work is not the first to tackle patient comfort by limiting the number of electrodes, whereas other authors have used three to six electrodes to experiment with different configurations^17,18,55^.

## Conclusion

This work concerns an extension and improvement of the application of EAs in seizure prediction. Our methodology proposes patient-specific seizure prediction models that perform above chance for 32% of the patients and may be used when the classical ML approach does not perform above chance or when we intend to retrieve interpretable insights. Additionally, these findings may evidence the need for different methodologies for different patients. Although this study includes 93 patients with several types of focal epilepsies and generalised epilepsy, the data concerns pre-surgical monitoring conditions. In these, due to time constraints in the clinic, patients suffer medication reduction and may suffer from sleep deprivation, which may induce more seizures that may not be representative of daily-life events^15^. To truly access seizure prediction performance, we must replicate this study on ultra long-term recordings collected during the patients’ daily life, such as the collected data from the Neurovista prediction challenge^35^ and in a proper field test. These two steps would answer how far this methodology may perform and how to apply it to a medical device at home. Concerning patient outcomes, we are aware of its limitations. In the future, we need to engage with patients and caregivers and focus on available technology, such as UNEEG SubQ^65,66^ and the ByteFlies Sensor Dots^67^. Patient-related outcome measures require a health-technology assessment^68,69^ which must be performed. Also, new software should meet hardware in a Value Sensitive Design approach^70^. Nevertheless, our work contributes to epilepsy seizure prediction by providing a complete pipeline for patient-specific prediction while addressing concerns regarding patient comfort in terms of electrode placement in the scalp.

The access to more computational resources may improve our methodology, for example, by enabling the increase of the number of features and/or the test of more complex classifiers. However, one must be careful with publication bias by reporting higher performances, as these may result from a test set optimisation. Also, these improvements will increase the execution time of the MOEA, which should be kept to a reasonable period. A higher runtime may imply the redefinition of some parameters, such as the number of generations and population size. Changing these aspects will not influence the application of the association rules, which can retrieve information about gene importance and gene interactions. We also believe our methodology may significantly benefit from information regarding concept drifts, such as medication intake and circadian cycles^14^.

## Supplementary Information

Supplementary Information.

## Data Availability

All code is available for public use in: https://github.com/MauroSilvaPinto/Interpretable-EEG-epilepsy-seizure-prediction-methods-using-multiobjective-evolutionary-algorithms.

## References

[1] Laxer KD (2014). The consequences of refractory epilepsy and its treatment. Epilepsy Behav..

[2] Fiest KM (2017). Prevalence and incidence of epilepsy: A systematic review and meta-analysis of international studies. Neurology.

[3] Ihle M (2012). EPILEPSIAE—A European epilepsy database. Comput. Methods Programs Biomed..

[4] Alvarado-Rojas C (2014). Slow modulations of high-frequency activity (40–140 Hz) discriminate pre-ictal changes in human focal epilepsy. Sci. Rep..

[5] Engel J (2016). What can we do for people with drug-resistant epilepsy? The 2016 Wartenberg Lecture. Neurology.

[6] Klatt J (2012). The EPILEPSIAE database: An extensive electroencephalography database of epilepsy patients. Epilepsia.

[7] Jette N, Engel J (2016). Refractory epilepsy is a life-threatening disease: Lest we forget. Neurology.

[8] Cloppenborg T (2016). Trends in epilepsy surgery: Stable surgical numbers despite increasing presurgical volumes. J. Neurol. Neurosurg. Psychiatry.

[9] Mormann F, Andrzejak RG, Elger CE, Lehnertz K (2007). Seizure prediction: The long and winding road. Brain.

[10] Gadhoumi K, Lina JM, Mormann F, Gotman J (2016). Seizure prediction for therapeutic devices: A review. J. Neurosci. Methods.

[11] Iasemidis LD (2003). Epileptic seizure prediction and control. IEEE Trans. Biomed. Eng..

[12] Bandarabadi M, Rasekhi J, Teixeira CA, Karami MR, Dourado A (2015). On the proper selection of preictal period for seizure prediction. Epilepsy Behav..

[13] Review A, Bou Assi E, Nguyen DK, Rihana S, Sawan M (2017). Towards accurate prediction of epileptic seizures. Biomed. Signal Process. Control.

[14] Kuhlmann L, Lehnertz K, Richardson MP, Schelter B, Zaveri HP (2018). Seizure prediction—Ready for a new era. Nat. Rev. Neurol..

[15] Freestone DR, Karoly PJ, Cook MJ (2017). A forward-looking review of seizure prediction. Curr. Opin. Neurol..

[16] Direito B (2012). Modeling epileptic brain states using EEG spectral analysis and topographic mapping. J. Neurosci. Methods.

[17] Direito B, Teixeira CA, Sales F, Castelo-Branco M, Dourado A (2017). A realistic seizure prediction study based on multiclass SVM. Int. J. Neural Syst..

[18] Teixeira C (2014). Epileptic seizure predictors based on computational intelligence techniques: A comparative study with 278 patients. Comput. Methods Programs Biomed..

[19] Moghim N, Corne DW (2014). Predicting epileptic seizures in advance. PLoS ONE.

[20] Park Y, Luo L, Parhi KK, Netoff T (2011). Seizure prediction with spectral power of EEG using cost-sensitive support vector machines. Epilepsia.

[21] Mirowski, P. W., LeCun, Y., Madhavan, D. & Kuzniecky, R. Comparing SVM and convolutional networks for epileptic seizure prediction from intracranial EEG. In *2008 IEEE Workshop on Machine Learning for Signal Processing* 244–249 (IEEE, 2008).

[22] Khan H, Marcuse L, Fields M, Swann K, Yener B (2017). Focal onset seizure prediction using convolutional networks. IEEE Trans. Biomed. Eng..

[23] Kiral-Kornek I (2018). Epileptic seizure prediction using big data and deep learning: Toward a mobile system. EBioMedicine.

[24] Abdelhameed, A. M. & Bayoumi, M. Semi-supervised deep learning system for epileptic seizures onset prediction. In *2018 17th IEEE International Conference on Machine Learning and Applications (ICMLA)* 1186–1191 (IEEE, 2018).

[25] Daoud H, Bayoumi MA (2019). Efficient epileptic seizure prediction based on deep learning. IEEE Trans. Biomed. Circuits Syst..

[26] Zhang Y, Guo Y, Yang P, Chen W, Lo B (2019). Epilepsy seizure prediction on eeg using common spatial pattern and convolutional neural network. IEEE J. Biomed. Health Inform..

[27] Wei X, Zhou L, Zhang Z, Chen Z, Zhou Y (2019). Early prediction of epileptic seizures using a long-term recurrent convolutional network. J. Neurosci. Methods.

[28] Schirrmeister RT (2017). Deep learning with convolutional neural networks for eeg decoding and visualization. Hum. Brain Mapp..

[29] Molnar, C. *Interpretable Machine Learning*, https://christophm.github.io/interpretable-ml-book/ (2019).

[30] Rudin C (2019). Stop explaining black box machine learning models for high stakes decisions and use interpretable models instead. Nat. Mach. Intell..

[31] Doshi-Velez, F. & Kim, B. Towards a rigorous science of interpretable machine learning. arXiv preprint arXiv:1702.08608 (2017).

[32] Goodman B, Flaxman S (2017). European union regulations on algorithmic decision-making and a “right to explanation. AI Mag..

[33] Cook MJ (2013). Prediction of seizure likelihood with a long-term, implanted seizure advisory system in patients with drug-resistant epilepsy: A first-in-man study. Lancet Neurol..

[34] Sun FT, Morrell MJ (2014). The rns system: Responsive cortical stimulation for the treatment of refractory partial epilepsy. Expert Rev. Med. Devices.

[35] Kuhlmann L (2018). Epilepsyecosystem.org: Crowd-sourcing reproducible seizure prediction with long-term human intracranial eeg. Brain.

[36] Pinto MF (2021). A personalized and evolutionary algorithm for interpretable eeg epilepsy seizure prediction. Sci. Rep..

[37] Baud MO, Proix T, Rao VR, Schindler K (2020). Chance and risk in epilepsy. Curr. Opin. Neurol..

[38] Jirsa VK, Stacey WC, Quilichini PP, Ivanov AI, Bernard C (2014). On the nature of seizure dynamics. Brain.

[39] Eiben AE, Smith JE (2003). What is an Evolutionary Algorithm?.

[40] Bartz-Beielstein T, Branke J, Mehnen J, Mersmann O (2014). Evolutionary algorithms. Wiley Interdiscip. Rev. Data Min. Knowl. Discov..

[41] Mitchell M, Taylor CE (1999). Evolutionary computation: An overview. Annu. Rev. Ecol. Syst..

[42] Borgelt, C. & Kruse, R. Induction of association rules: Apriori implementation. In *Compstat* 395–400 (Springer, 2002).

[43] Dreifuss FE (1998). A comparison of rectal diazepam gel and placebo for acute repetitive seizures. N. Engl. J. Med..

[44] Liu H, Motoda H (1998). Feature Extraction, Construction and Selection: A Data Mining Perspective.

[45] Motoda, H. & Liu, H. Feature selection, extraction and construction. In *Communication of IICM*, Vol. 5 (Institute of Information and Computing Machinery) 2 (2002).

[46] Sondhi P (2009). Feature construction methods: A survey. Sifaka. Cs. Uiuc. Edu.

[47] Rasekhi J, Mollaei MRK, Bandarabadi M, Teixeira CA, Dourado A (2013). Preprocessing effects of 22 linear univariate features on the performance of seizure prediction methods. J. Neurosci. Methods.

[48] Bulusu, S., Prasad, R. S. S. S., Telluri, P. & Neelima, N. Methods for epileptic seizure prediction using eeg signals: A survey. In *Artificial Intelligence Techniques for Advanced Computing Applications* 101–115 (Springer, 2021).

[49] Stacey W (2020). Emerging roles of network analysis for epilepsy. Epilepsy Res..

[50] Kramer MA, Cash SS (2012). Epilepsy as a disorder of cortical network organization. Neuroscientist.

[51] Kramer MA (2010). Coalescence and fragmentation of cortical networks during focal seizures. J. Neurosci..

[52] Jacobs D, Hilton T, Del Campo M, Carlen PL, Bardakjian BL (2018). Classification of pre-clinical seizure states using scalp eeg cross-frequency coupling features. IEEE Trans. Biomed. Eng..

[53] Bai Y, Liang Z, Li X (2015). A permutation Lempel-Ziv complexity measure for eeg analysis. Biomed. Signal Process. Control.

[54] Jia X, Kohn A (2011). Gamma rhythms in the brain. PLoS Biol..

[55] Bandarabadi M, Teixeira CA, Rasekhi J, Dourado A (2015). Epileptic seizure prediction using relative spectral power features. Clin. Neurophysiol..

[56] Deb K, Pratap A, Agarwal S, Meyarivan T (2002). A fast and elitist multiobjective genetic algorithm: Nsga-II. IEEE Trans. Evol. Comput..

[57] Cormen, T. H., Leiserson, C. E., Rivest, R. L. & Stein, C. Introduction to algorithms second edition. In *The Knuth-Morris-Pratt Algorithm, Year* (2001).

[58] Teixeira, C., Direito, B., Bandarabadi, M. & Dourado, A. Output regularization of SVM seizure predictors: Kalman filter versus the firing power method. In *Proceedings of the Annual International Conference of the IEEE Engineering in Medicine and Biology Society, EMBS* 6530–6533. 10.1109/EMBC.2012.6347490 (2012). 10.1109/EMBC.2012.6347490 23367425

[59] Andrzejak RG (2003). Testing the null hypothesis of the nonexistence of a preseizure state. Phys. Rev. E Stat. Phys. Plasmas Fluids Relat. Interdiscip. Top..

[60] Winterhalder M (2003). The seizure prediction characteristics: A general framework to assess and compare seizure prediction methods. Epilepsy Behav..

[61] Gramfort A (2013). MEG and EEG data analysis with MNE-Python. Front. Neurosci..

[62] Okamoto M (2004). Three-dimensional probabilistic anatomical cranio-cerebral correlation via the international 10–20 system oriented for transcranial functional brain mapping. Neuroimage.

[63] Schelter B (2006). Testing statistical significance of multivariate time series analysis techniques for epileptic seizure prediction. Chaos.

[64] Schulze-Bonhage A (2010). Views of patients with epilepsy on seizure prediction devices. Epilepsy Behav..

[65] Weisdorf S (2019). Ultra-long-term subcutaneous home monitoring of epilepsy-490 days of eeg from nine patients. Epilepsia.

[66] Duun-Henriksen J (2020). A new era in electroencephalographic monitoring? Subscalp devices for ultra-long-term recordings. Epilepsia.

[67] Nasseri M (2020). Signal quality and patient experience with wearable devices for epilepsy management. Epilepsia.

[68] Marras CE (2013). Health technology assessment report on the presurgical evaluation and surgical treatment of drug-resistant epilepsy. Epilepsia.

[69] Whiting P (2006). A systematic review of the effectiveness and cost-effectiveness of neuroimaging assessments used to visualise the seizure focus in people with refractory epilepsy being considered for surgery. Health Technol. Assess. (Winchester, England).

[70] Van Andel J, Leijten F, Van Delden H, van Thiel G (2015). What makes a good home-based nocturnal seizure detector. PLoS ONE.

