# Supplementary material for: Interpretable EEG seizure prediction using a multiobjective evolutionary algorithm
Source: Sci Rep. 2022 Mar 15;12:4420. doi: 10.1038/s41598-022-08322-w (PMC8924190; doi:10.1038/s41598-022-08322-w)
Supplement: Supplementary file 1 — Supplementary Information. [file 41598_2022_8322_MOESM1_ESM.pdf]

Interpretable EEG seizure prediction using a multiobjective evolutionary algorithm

Mauro Pinto[1,+,*], Tiago Coelho[1,+], Adriana Leal[1], Fábio Lopes[1,2], António Dourado[1], Pedro Martins[1], and César Teixeira[1]

[1]Univ Coimbra, CISUC, Department of Informatics Engineering, Coimbra, Portugal
[2]Epilepsy Center, Department Neurosurgery, Medical Center - University of Freiburg, Faculty of Medicine, University of Freiburg, Freiburg, Germany
[*]mauropinto@dei.uc.pt
[+]these authors contributed equally to this work

Supplementary Material

Patient data

Patient ID	Hospital	Sex	Age	Fs (Hz)	#Seiz. (Train/ Test)	Rec. duration (hours)	Focus loc.	Surgery decision	Seizure activity pattern	Seizure classification	Vigilance state at seizure onset
102	UKLFR	m	36	256	3<br>8	12.00<br>109.36	c-l, p-r	i-s	t, t, t /<br>d, t, t, t, t, d,<br>t, t	FOIA, FOIA, UC /<br>FOIA, UC, FOIA, FBTC, FOIA, FOIA<br>FOIA, FOIA	A, 2, A /<br>A, A, A, A, 2,<br>A, 2, A
202	UKLFR	m	24	256	3<br>1	12.00<br>22.12	–l	s	b, r, r<br>t	FOIA, FOIA, FOIA<br>FOIA	R, A, 2<br>2
500	HUC	m	57	512	3<br>4	12.00<br>66.39	t-r	s	a, a, a<br>a, a, a, a	FOIA, FOIA, FOIA<br>FOIA, FOIA, FOIA, UC	A, A, A<br>A, A, A, A
1200	HUC	f	40	1024	3<br>1	12.00<br>11.44	t-r	o	t, a, a<br>a	FOIA, UC, FOIA<br>UC	A, A, A<br>A
1500	HUC	m	50	1024	3<br>1	12.00<br>6.91	t-r	o	t, t, t<br>t	FOIA, FOIA, FOIA<br>FOIA	3, A,3<br>A
1600	HUC	m	33	512	3<br>1	12.00<br>10.87	t-l	o	a, a, a<br>a	FOIA, UC, FOIA<br>FOIA	2, 3, A<br>A
2100	HUC	m	20	512	3<br>3	12.00<br>36.16	t-l	o	t, t, t<br>t, t, t	UC, FOIA, FOIA<br>FOIA, FOIA, FOIA	2, A, A<br>3, A, A
2300	HUC	m	49	512	3<br>1	12.00<br>7.13	t-l	o	t, t, t<br>t	FOA, UC, UC<br>FOA	A, 1, A<br>A
3300	HUC	m	29	1024	3<br>1	12.00<br>8.50	-	o	t, d, d<br>t	UC, UC, FOA<br>UC	A, 2, A<br>A
4500	HUC	m	49	1024	3<br>1	12.00<br>14.62	t-l	o	t, a, a<br>a	FOIA, FOIA, FOIA<br>FOIA	A, A, A<br>A
5800	HUC	f	26	1024	3<br>2	12.00<br>18.35	t-b	o	a, a, a<br>a, a	UC, UC, FOIA<br>UC, FOIA	A, A, A<br>A, A
6000	HUC	m	16	512	3<br>1	12.00<br>48.71	t-l	o	s, a, a<br>d	UC, UC, FBTC<br>UC	A, 2, 3<br>2
7200	HUC	m	41	1024	3<br>1	12.00<br>4.17	t-b	-	b, b, d<br>d	FBTC, FBTC, FBTC<br>FBTC, FBTC	2, 2, 2<br>2
8100	HUC	m	16	1024	3<br>2	12.00<br>45.29	t-b	n	b, b, b<br>b, s	FOIA, FOIA, UC<br>FOIA, UC	A, A, A<br>A, 1
8902	UKLFR	f	67	256	3<br>2	12.00<br>22.48	tpl	n	a, b, a<br>m, a	UC, FOIA, FOIA<br>FOIA, FOIA	A, A, A<br>A, A
11002	UKLFR	m	41	256	3<br>1	12.00<br>1.69	tpr	s	?, s, a<br>t	UC, FOIA, FOIA<br>FOIA	A, R, A<br>A
11502	UKLFR	m	34	256	3<br>10	12.00<br>120.40	tmr	s	b, b, b /<br>b, a, ?, t, s,<br>b, b, b, b, b	FOIA, FOA, FOIA /<br>FOIA, FOA, FOA, FOA, UC,<br>UC, FOA, FOIA, FOIA, FOIA	2, A, A /<br>A, A, ?, A, 1,<br>A, A, A, A, A

Patient ID	Hospital	Sex	Age	Fs (Hz)	#Seiz. (Train/ Test)	Rec. duration (hours)	Focus loc.	Surgery decision	Seizure activity pattern	Seizure classification	Vigilance state at seizure onset
12702	UKLFR	f	16	256	3<br>3	12.00<br>67.50	tbl, –b, tll	o	r, r, r<br>l, l, l	FOIA, UC, FOIA<br>UC, FOA, FOIA	A, 2, A<br>2, A, 2
21602	UKLFR	m	44	256	3<br>6	12.00<br>57.75	tpr, f-l	o	?, ?, b /<br>t, b, s, b, d, b	FOA, UC, UC<br>UC, UC, UC, UC, UC, UC	A, A, A/<br>2, A, 2, A, R, A
21902	UKLFR	m	47	256	3<br>1	12.00<br>9.76	t-l	s	t, t, t<br>b	UC, FOIA, FOIA<br>FOIA	A, A, A<br>R
22602	UKLFR	m	26	256	3<br>2	12.00<br>22.03	f-l	i-n	b, b, ?<br>b, b	UC, UC, UC<br>UC, UC	A, A, A<br>2, A
23902	UKLFR	m	36	256	3<br>2	12.00<br>33.92	t-l	s	t, t, t<br>d, t	FOA, FOA, FOA<br>FOA, FOA	A, A, A<br>A, A
26102	UKLFR	m	65	256	3<br>1	12.00<br>22.56	t-l	i-n	m, t, t<br>t	FOIA, FOIA, FOIA<br>FOIA	A, A, A<br>A
30802	UKLFR	m	28	256	3<br>5	12.00<br>61.67	t-r, t-l	o	t, t, t<br>t, t, t, t, t	FOA, FOA, FOA<br>FOA, FOA, FOA, FOA, FOA	R, A, 2<br>A, A, R, 2, 2
32502	UKLFR	m	46	256	3<br>3	12.00<br>29.01	t-b, f-r	i-n	t, t, t<br>t, t, t	FOIA, FBTC, UC<br>UC, FBTC, FBTC	A, A, A<br>A, 2, A
46702	UKLFR	f	15	256	3<br>2	12.00<br>12.88	tmr	o	a, a, t<br>b, t	FOA, FOIA, FOIA<br>FBTC, FOIA	A, 2, A<br>2, A
50802	UKLFR	m	43	256	3<br>2	12.00<br>35.58	t-l	i-n	t, t, t<br>t, t	FOIA, UC, UC<br>FOIA, FBTC	A, 2, 2<br>2, A
51002	UKLFR	f	46	256	3<br>5	12.00<br>47.10	f-r, t-l	s	?, a, a<br>t, t, t, t, t	FOA, FOIA, FOIA<br>FOIA, FOIA, FOIA, UC, FOIA	A, A, A<br>A, A, A, A, A
52302	UKLFR	f	61	256	3<br>1	12.00<br>6.84	t-l	i-s	?, ?, d<br>t	UC, FOA, UC<br>UC	A, A, 1<br>A
53402	UKLFR	m	39	256	3<br>2	12.00<br>50.45	t-l, t-r	s	?, ?, ?<br>?, t	FOA, FOA, FOA<br>FOA, FOIA	A, A, 2<br>A, A
55202	UKLFR	f	17	256	3<br>5	12.00<br>65.32	t-r, t-b	i-n	t, d, t<br>t, t, t, r, r	FOIA, FOIA, FOA<br>UC, UC, FOA, UC, FOIA	A, A, A<br>A, A, A, A, A
56402	UKLFR	m	47	256	3<br>1	12.00<br>20.23	t-r, t-l	n	t, ?, ?<br>a	UC, UC, UC<br>FBTC	A, A, A<br>A, A
58602	UKLFR	m	32	256	3<br>3	12.00<br>23.33	t-l	i-n	r, t, t<br>r, r, t	FOIA, FOIA, FOIA<br>FOIA, FOIA, FOIA	A, R, A<br>A, A, 2
63502	UKLFR	f	63	1024	3<br>2	12.00<br>28.25	tml, t-r	o	s, s, s<br>s, s	FOIA, UC, FOIA<br>FOIA, FOIA	A, A, A<br>2, 2
64702	UKLFR	m	51	256	3<br>2	12.00<br>31.60	tmr	i-s	?, m, t<br>t, t	FOA, FBTC, FBTC<br>FBTC, FBTC	A, A, A<br>A, 2

Patient ID	Hospital	Sex	Age	Fs (Hz)	#Seiz. (Train/ Test)	Rec. duration (hours)	Focus loc.	Surgery decision	Seizure activity pattern	Seizure classification	Vigilance state at seizure onset
70302	UKLFR	m	18	2500	3<br>7	12.00<br>88.85	tpr	s	s, ?, a /<br>s, b, s, s, s,<br>s, s	FBTC, FOIA, UC /<br>UC, FOA, UC, FBTC, FBTC,<br>UC, FBTC	2, 2, A /<br>A, A, 2, A, 2,<br>2, 2
70902	UKLFR	f	28	2500	3<br>2	12.00<br>17.87	-	n	?, ?, t<br>?, t	FOA, FOA, FOA<br>FOA, FBTC	A, A, A<br>A, A
71102	UKLFR	f	31	2500	3<br>2	12.00<br>16.65	t-r, t-l	n	t, b, t<br>r, r	FBTC, FBTC, FOA<br>FOA, FOA	2, A, A<br>A, A
71502	UKLFR	f	18	2500	3<br>2	12.00<br>31.18	–l, t-r	n	t, t, d<br>t, ?	FOIA, FOA, UC<br>SP, FBTC	2, 2, 2<br>2, 2
71802	UKLFR	f	46	2500	3<br>2	12.00<br>33.76	-	n	t, t, t<br>?, t	FOIA, FOIA, UC<br>FOA, FOIA	A, A, A<br>A, A
73002	UKLFR	m	32	2500	3<br>2	12.00<br>14.77	t-r, t-l	n	d, ?, t<br>t, t	FBTC, FBTC, FBTC<br>FOIA, UC	A, A, A<br>R, A
75102	UKLFR	f	2	256	3<br>2	12.00<br>26.97	-	n	t, a, t<br>?, t	UC, UC, UC<br>UC, UC	2, 2, 2<br>A, 2
75202	UKLFR	m	13	256	3<br>4	12.00<br>52.62	c-r, o-r,<br>t-r	s	t, t, t<br>t, t, ?, t	FOIA, FOIA, UC<br>FOIA, FOIA, FOIA, FOIA	2, 2, A<br>A, A, A, A
79502	UKLFR	f	35	256	3<br>4	12.00<br>41.63	p-l, t-l	i-n	s, b, r<br>b, p, b b	FOIA, FOIA, FOIA<br>FOIA, FOIA, FOIA, FOIA	A, A, A<br>A, A, A, A
80602	UKLFR	f	34	256	3<br>2	12.00<br>88.14	f-l	i-n	?, ?, r<br>?, ?	FOIA, FOIA, FBTC<br>FOIA, FOIA	A, A, A<br>A , A
80702	UKLFR	f	22	256	3<br>3	12.00<br>29.53	t-b	n	b, b, ?<br>c, c, c	FOIA, FOIA, UC<br>FOIA, FBTCB, FOIA	A, A, A<br>A, A, A
81402	UKLFR	f	65	256	3<br>3	12.00<br>60.59	f-l	n	t, ?, t<br>t, t, d	UC, FOA, UC<br>FOIA, FOIA, FOIA	A, A, 2<br>2, 2, A
85202	UKLFR	f	54	256	3<br>2	12.00<br>20.41	t-l	s	m, c, m<br>m, m	FOIA, FOIA, UC<br>UC, UC	2, A, A<br>A, A
92102	UKLFR	m	38	256	3<br>2	12.00<br>84.74	plr	s	r, r, ?<br>r, r	FOIA, UC, FOIA<br>FOIA, FOIA	A, A, A<br>A, A
93402	UKLFR	m	67	256	3<br>2	12.00<br>54.03	tpl	n	t, t, t<br>t, t	FBTC, FOIA, FOIA<br>UC, UC	2, 2, 2<br>2, 2
93902	UKLFR	m	50	256	3<br>3	12.00<br>20.27	t-r	i-n	t, t, d<br>d, d, d	FOA, FOIA, FBTC<br>FOIA, FOIA, UC	A, A, 2<br>A, 2, A
94402	UKLFR	f	37	256	3<br>4	12.00<br>30.65	t-r	o	?, d, b<br>t, ?, b, ?	FOA, UC, FOIA<br>UC, FOA, UC, FOA	A, A, A<br>2, A, 2, A
95202	UKLFR	f	50	256	3<br>4	12.00<br>90.25	t-l	s	b, b, b<br>m, b, b, t	FBTC, FOIA, FOIA<br>FOIA, UC, FOIA, UC	2, 2, 2<br>2, 2, 2, 2

Patient ID	Hospital	Sex	Age	Fs (Hz)	#Seiz. (Train/ Test)	Rec. duration (hours)	Focus loc.	Surgery decision	Seizure activity pattern	Seizure classification	Vigilance state at seizure onset
96002	UKLFR	m	58	256	3<br>4	12.00<br>82.14	t-l, t-r	s	t, t, t<br>d, a, t, a	FOIA, FOIA, FOIA, FOIA<br>UC, FOIA, FOIA	A, A, A<br>A, A, A, A
98102	UKLFR	m	36	256	3<br>2	12.00<br>45.73	t-l	i-n	?, ?, ?<br>?, ?	FOA, UC, UC<br>UC, FBTC	A, A, A<br>A, A, A, A
100002	UKLFR	m	35	256	3<br>6	12.00<br>72.19	-	o	?, t, ? /<br>t, t, c, c, ?,<br>c	FOA, FOA, FOA /<br>FOA, FOIA, FOA, UC, FOA,<br>UC	A, A, A /<br>A, 2, A, 1, A,<br>2
101702	UKLFR	m	52	256	3<br>2	12.00<br>23.81	t-r, t-l	n	t, t, t<br>r, r	FOIA, FOIA, FOIA<br>FOIA, FOIA	A, A, A<br>2, A
102202	UKLFR	m	17	256	3<br>4	12.00<br>51.39	t-l, tpl	n	b, ?, t<br>?, t, t, t	FOA, UC, FOIA<br>UC, FOA, FOIA, UC	2, A, 2<br>A, A, 2, A
103002	UKLFR	f	31	256	3<br>3	12.00<br>92.49	o-r	o	t, d, ?<br>a, r, b	FOIA, FOIA, FOIA<br>UC, FOIA, UC	A, A, A<br>A, A, A
103802	UKLFR	f	17	256	3<br>3	12.00<br>19.64	-	-	r, ?, t<br>t, t, t	FOIA, FOA, FOIA<br>FOIA, FOIA, FOIA	2, A, A<br>1, A
104602	UKLFR	f	17	256	3<br>2	12.00<br>15.24	tml	o	t, a, t<br>t, d	FOIA, FBTC, FBTC<br>FBTC, UC	A, 2, 2<br>2, 2
109202	UKLFR	f	49	256	3<br>1	12.00<br>7.20	f-l, f-r	n	?, a, a<br>b	FOIA, FBTC, FBTC<br>FBTC	A, 2, R<br>2
109502	UKLFR	m	50	256	3<br>1	12.00<br>41.91	t-l, t-r	n	t, t, t<br>t	FOIA, FOIA, UC<br>UC	A, A, A<br>A
109602	UKLFR	f	32	1024	3<br>1	12.00<br>21.29	tml	s	b, r, b<br>b	FOIA, FOIA, FOIA<br>UC	A, A, A<br>A
110602	UKLFR	m	56	256	3<br>2	12.00<br>25.90	t-r	o	t, t, t<br>t, t	FOIA, FOIA, FOIA<br>FOIA, FOA	A, A, A<br>A, A
111902	UKLFR	m	42	256	3<br>2	12.00<br>14.57	c-l	n	d, t, t<br>s, t	FBTC, UC, FBTC<br>FBTC, FBTC	A, A, A<br>A, A
112402	UKLFR	f	32	256	3<br>9	12.00<br>61.80	f-r	i-s	d, t, ? /<br>b, b, b, d, t,<br>b, s, b, t	FOIA, FOA, FOIA /<br>FOIA, FOIA, FOIA, FBTC, FOIA,<br>FOIA, FOA, UC, FOIA	A, A, A /<br>2, A, A, A, A,<br>A, A, A 1
113902	UKLFR	f	29	256	3<br>3	12.00<br>22.73	t-r	o	t, d, t<br>t, t, t	UC, FOIA, FOIA<br>FOIA, UC, FOIA	A, A, 2<br>A, 2 A
114702	UKLFR	f	22	256	3<br>5	12.00<br>34.02	tpr	o	t, t, t<br>t, d, t, d, t	FOIA, FOIA, UC<br>FOIA, FOIA, FOIA, FOIA, FOIA	A, A, A<br>A, A, A, A, A
114902	UKLFR	f	16	256	3<br>4	12.00<br>50.63	t-r, tll	n	s, b, s<br>t, r, a, t	FOA, FOIA, FOIA<br>FBTC, UC, FOIA, FOIA	A, A, A<br>2, A, A, A
115202	UKLFR	f	32	256	3<br>4	12.00<br>70.96	o-r, t-l	n	t, t, t<br>t, b, b, b	FOIA, UC, FOIA<br>FOIA, FOIA, FOIA, UC	2, 2, 2<br>2, 2, 2, 2

Patient ID	Hospital	Sex	Age	Fs (Hz)	#Seiz. (Train/ Test)	Rec. duration (hours)	Focus loc.	Surgery decision	Seizure activity pattern	Seizure classification	Vigilance state at seizure onset
123902	UKLFR	f	25	256	3<br>2	12.00<br>30.15	t-l, t-r	i-n	t, t, t<br>t, t	FBTC, FBTC, FOIA<br>FOIA FOA	2, 2, R<br>A, A
125002	UKLFR	m	39	256	3<br>4	12.00<br>39.28	-	n	?, ?, ?<br>?, ?, ?, a	UC, FOIA, UC<br>FOIA, FOIA, UC, UC	A, A, A<br>A, 2, A, A
1235103	HdlPS	m	19	400	3<br>2	12.00<br>34.71	t-b	n	r, l, l<br>l, l	UC, UC, UC<br>UC, FBTC	3, A, ?<br>3, 3
1307103	HdlPS	f	37	512	3<br>5	12.00<br>191.76	t-b, t-l,<br>h-l	o	l, l, l<br>l, s, t, t, l	FOIA, FOIA, FOIA<br>FOIA, FOIA, FOIA, FOIA, FOIA	A, A, 1<br>A, A, A, A, A
1308503	HdlPS	m	19	512	3<br>1	12.00<br>8.98	h-l, t-l	-	s, l, l<br>l	FOIA, FOIA, UC<br>FOIA	2, A, A<br>2
1308603	HdlPS	m	35	512	3<br>1	12.00<br>64.49	t-b, t-r	-	t, t, t<br>m	UC, FOIA, FOIA<br>FOIA	1, A, A<br>A
1310803	HdlPS	f	38	512	3<br>1	12.00<br>22.28	tml	o	t, t, t<br>t	FOIA, FOIA, FOIA<br>FOIA	?, ?, A<br>A
1312903	HdlPS	f	19	512	3<br>1	12.00<br>48.01	fll	i-n	a, t, a<br>t	FOIA, FOIA, UC / UC	2, A, 1<br>1
1315203	HdlPS	m	21	512	3<br>1	12.00<br>34.81	t-r, f-r	o	l, d, t<br>t, t	UC, FOIA, UC / FOIA	R, A, R<br>A
1319203	HdlPS	m	28	400	3<br>4	12.00<br>65.70	–r	n	l, l, m<br>m, a, a, a	UC, UC, UC<br>FOIA, FOIA, UC, FOA	A, 1, A<br>2, A, A, A
1321803	HdlPS	f	27	512	3<br>1	12.00<br>4.17	tmr, tmb,<br>tml	i-n	t, t, d<br>d	UC, FOIA, FOIA<br>FOIA	3, 2, A<br>2
1322803	HdlPS	m	18	400	3<br>1	12.00<br>22.11	t-r, c-r	i-n	t, t, t<br>t	FOIA, FOIA, FBTC<br>UC	A, A, A<br>A
1324903	HdlPS	f	22	400	3<br>2	12.00<br>17.43	c-r	o	l, l, l<br>l, l	UC, UC, FOA<br>UC, UC	1, 1, 1<br>A, A
1325103	HdlPS	f	37	400	3<br>1	12.00<br>23.19	t-r	o	a, l, l<br>l	FBTC, FOIA, UC<br>FOIA	2, 2, 2<br>R
1325403	HdlPS	m	37	400	3<br>1	12.00<br>6.61	tpl	i-n	l, l, a<br>l	FOIA, FOIA, FOIA<br>FOIA	A, A, A<br>A
1325603	HdlPS	f	37	400	3<br>1	12.00<br>4.51	t-l, t-r	n	a, t, t<br>a	FOIA, FOIA, FOIA<br>FBTC	A, A, A<br>2
1326103	HdlPS	f	34	400	3<br>1	12.00<br>22.39	t-r	s	a, r, r<br>r	FOA, UC, UC<br>UC	A, 2, 2<br>2
1327403	HdlPS	m	50	400	3<br>1	12.00<br>9.27	t-l	i-n	m, t, d<br>d	UC, UC, FBTC<br>UC	A, 2, 2<br>1
1328603	HdlPS	m	42	400	3<br>1	12.00<br>11.84	tml	s	t, l t<br>t	UC, FOA, UC<br>UC	2, A, A<br>A

Patient ID	Hospital	Sex	Age	Fs (Hz)	#Seiz. (Train/ Test)	Rec. duration (hours)	Focus loc.	Surgery decision	Seizure activity pattern	Seizure classification	Vigilance state at seizure onset
1328803	HdlPS	m	46	400	3<br>1	12.00<br>12.75	f-r	i-n	c, l, l<br>c	UC, UC, UC<br>UC, UC	A, A, A<br>A
1328903	HdlPS	m	32	400	3<br>2	12.00<br>154.72	t-b	o	l, d, l<br>m, r	FOA, FOA, FOA<br>FOA, FOA	A, A, A<br>A, A
1330903	HdlPS	m	36	400	3<br>5	12.00<br>111.07	h-b, h-l	s	l, t, l<br>l, d, t, t, l	FOIA, FOIA, FOIA<br>FOIA, FOIA, FOIA, FOIA, FOIA	A, A, A<br>A, A, A, A, A

Table S 1. Patient information. For each patient, it is presented its EPILEPSIAE ID, the number of seizures used for training and testing and their recording duration, seizure focus (as lobe-subegion-side, frontal lobe (f), temporal lobe (t), central lobe (c), occipital lobe (o), parietal lobe (p), mesial subregion (m), basal subregion (b), polar subregion (p), right side (r), left side (l), and bilateral (b)), the annotated activity pattern (unclear (?), rhythmic sharp waves (s), rhythmic alpha waves (a), rhythmic delta waves (d), rhythmic theta waves (t), rhythmic beta waves (b), repetitive spiking (r), cessation of inter-ictal activity (c), amplitude depression (m), low amplitude fast activity (l)), seizure classification (unclassified (UC), Focal Onset Aware (FOA), Focal Onset Impaired Awareness (FOIA), Focal to Bilateral Tonic-Clonic (FBTC)), sleep stage at onset (awake (A), sleep stage I (1), II (2), III (3), IV (4) and REM (R)), and surgery decision (performed (s), offered but not performed (o), not offered (n), and invasive monitoring required (i)).

Features Description

We justify here the use of our features: statistical moments, Hjorth parameters, relative spectral power, wavelet coefficient energy, and spectral edge frequency. We only used linear features. These are mathematical techniques that use phase/frequency and amplitude information of the signal and comply with the linearity property. When one extracts linear features, assumes the quasi-stationarity of the EEG signal within each time window from the data segmentation step in the signal pre-processing stage.

Statistical Moments

Several studies[1–8] report the use of statistical moments for characterising the amplitude distribution of the EEG time series. The first moment is the mean, the second moment is the variance, the third moment is the skewness, and the fourth moment is the kurtosis. The skewness is zero for symmetric amplitude distributions and non-zero for asymmetric distributions. The kurtosis measures the relative peakedness or flatness of an amplitude distribution[2]. These statistical measures have shown significant changes during the pre-ictal period compared to the inter-ictal state[3,5]. A decrease in the variance and an increase in the kurtosis were observed in the pre-ictal period when compared with inter-ictal based data[4,9].

Hjorth Parameters

Hjorth parameters are three time-domain measures of brain activity that authors have been using in seizure anticipation. These parameters have the intention of constituting a clinically helpful tool for quantitatively describing the electroencephalogram (EEG)[2]. The activity, mobility, and complexity are measures of mean power, root-mean-squared frequency, and root-mean-square frequency spread, respectively. Authors have reported a significant increase in the mobility and complexity of the EEG during the pre-ictal period[3,5].

Relative Spectral Power

Different physiological and pathological processes are reflected by activity in different frequency ranges of the power spectrum of the EEG[2]. Relative spectral power band can be calculated by firstly computing the Power Spectral Density (PSD) of the time-series within a time window. It is essential to mention that the calculation of the PSD assumes the signal in each window is short enough to be considered quasistationary and long enough to capture the brain low-frequency activity. Although there are several methods for spectral estimation, the simplest form can be by applying the Fast Fourier Transform (FFT) and then averaging its squared coefficients that belong to the frequency band of interest. A normalised spectral power provides a more robust measure of the fluctuations in a patient's daily life to reveal the pre-ictal state[3]. Authors have reported that brain activity increases or decreases at specific frequency bands during certain events. Before seizure onset, there may be a transfer of power from the lower to the higher frequencies[3–5,10–12]. For example, Mormann et al.[5] showed a decrease in Delta band power, coupled with a relative power decrease in the other sub-bands. Bandarabadi et al.[10] showed that relative combinations of sub-band spectral powers across channel pairs might be used for tracking gradual changes preceding seizures.

Wavelet Coefficients Energy

Wavelet transform is a time-frequency domain transform that can be an alternative to the FFT as it decomposes the signal in different resolution levels according to different frequency ranges[1,3,4]. In other words, wavelets provide a time-variant decomposition adapted to the signal, capturing minor details and sudden changes by providing higher frequency resolution for lower frequencies and higher time resolution to higher frequencies. With the signal decomposed, it is possible to compute several measures using the wavelet coefficients, as in the case of signal energy. By computing the energy of the signals originated by the decomposition, a measure of the energy in different frequency ranges can be achieved[3,4,13].

Spectral Edge Frequency

Usually, most EEG signal's power is within the frequency band from 0 Hz up to 40Hz[2]. Spectral edge frequency quantifies the power contained in lower frequencies concerning a given threshold. Spectral edge frequency (SEF) is the measure that indicates the frequency below x per cent of the overall signal power is contained and is frequently used in seizure prediction[1,3–5]. As the existence of a power transfer from low to high frequencies has been reported during the pre-ictal stage, SEF may also be capable of capturing these dynamics.

Multiobjective Evolutionary Algorithm Details (MOEA) Configuration Details

Once every individual has been evaluated, parents are selected to reproduce and generate offspring. In this study, considering a population of N individuals, a group of $N/2$ parents are selected and recombine until N offspring are produced. The offspring is then subjected to mutation (with a given probability, just as before for recombination) and a replacement strategy is put in place to select the N individuals that will make up the following generation. To do these steps, we firstly need to rank the population, where we used non-donimated sorting.

For each individual, its rank is equal to the number of individuals dominating it plus one (e.g. all nondominated individuals are assigned rank 1). Afterwards, a fitness value was assigned by interpolating from the best individual (rank 1) to the worst (rank $n \leq N$) within the individuals of each rank. In other words, we iteratively searched for all the non-dominated solutions in the population that have not been labelled as belonging to a previous front. After labelling that new front, a front counter was incremented and the process was repeated until all solutions have been ranked. Afterwards the individuals within each rank were sorted according to a measure, the crowding distance, which corresponds to the average side length of a cuboid defined by its nearest neighbours in the same front[14]. In essence, the larger the crowding distance is, the fewer solutions occupy the vicinity of a given individual. It is computed, for each objective m, in the following manner: sort individuals according to their fitness score f, assign an infinite value to boundary solutions (so that they are always selected), and compute the distance measure for the remaining solutions as given by Eq. 1. The overall crowding distance was calculated as the sum of the distance values concerning each objective.

$$F(i) = \frac{f(i+1)_m - f(i-1)_m)}{f_m^{max} - f_m^{min}} \tag{1}$$

For parent selection, the population was ranked using non-dominated sorting and then, within each rank, individuals were sorted by the crowding distance (the higher it was, the better rank they were assigned to). Parents were then chosen using binary tournaments and reproduced until N offspring were generated. With respect to the replacement strategy, we used an elitist approach. Firstly, after evaluating the newly created offspring, the $2N$ individuals (current generation and offspring) were ranked with non-dominated sorting. Then, entire fronts were added into the new generation, starting from the rank 1 individuals, followed by rank 2, and so on, until a new set could no longer be accommodated. This last set of solutions was then sorted according to the crowding distance, and the better ones were chosen to fill out the rest of the new population.

Neighbourhood details

We provide here more details concerning the established neighbourhoods. As in Pinto et al.[15], window length and time instant genes have a straightforward ordering, as their values correspond to increasing/decreasing discrete time intervals. Electrodes' neighbourhood is based on their scalp position. As there is no decreasing/increasing relationship among the mathematical operators (mean, median, variance, integral), all genes were considered neighbours of each other. In this work, the difference is in the feature's genes, as significantly more options are available.

The extracted features, however, can be divided into several groups and sub-groups, as seen in Fig. 3.b). Firstly, they can be divided concerning their time/frequency domain. Then, time-domain features can be divided into statistical moments (ordered by their *n*-th moment: mean, variance, skewness, kurtosis) or into Hjorth Parameters (ordered by their *n*-th moment: activity, mobility, complexity). Frequency-domain features can be divided into spectral edge features (ordered by the frequency: 50%, 75%, and 90%) or frequency band ones. Frequency band features can be divided into relative spectral power bands (ordered by their frequency range: delta, theta, beta, alpha, low-gamma, high-gamma) and the energy from wavelet decomposition levels (ordered by their decomposition levels: D1, D2, D3, D4, D5, D6, D7, and D8). Please note that the used overall division might lead to discussion, as wavelet transforms may be considered forms of time-frequency representations and not solely belonging to the frequency-domain. Nevertheless, we justify our choice as we believe that these features are conceptually similar to relative power spectral bands.

Control method

We used a control methodology to verify the performance of our approach, inspired in the existing clinical trial that deals with EEG seizure susceptibility detection, namely the Seizure Advisory System Feasibility Study (NCT01043406)[16] and other previous works, such as Direito et al.[1]. To simplify the process, its development was merely inspired and not fully reproduced.

The Neurovista Advisory System used a patient-specific layered structure, which includes filtering, feature extraction, and classification. Input signals were filtered by a set of octave-wide digital filters, from 8Hz to 128Hz range. Notch filters were also optionally available. The signals were firstly segmented where they extracted the average energy, Teager-Kaiser energy, and line-length, into windows of 5 seconds and, secondly, these features were normalised. This output resulted in a total of 288 features, as these resulted from the combination of 16 available iEEG input channels, 6 filter/normalisation options, and 3 available features. During training, the 16 best performing features are selected by using a backward elimination feature selection method based on Hilbert-Schmidt Independence Criterion (BAHSIC). The selected features were used to training a classification model, which was inspired by two types of classification models, decision trees and a k-nearest neighbors (kNN). In this, a given feature vector was classified by using several decision surfaces. Simply put, the input was firstly compared to the first decision surface to determine the binary side of the surface where the point was on. Then, and depending on the previous binary answer, a new decision surface is selected to perform a second surface comparison. This process continues until reaching a total of ten decision layers. Thus, the feature space was divided in 2^{10} partitions. The used surfaces were chosen during training. To each, it was assigned a relative measure of seizure risk concerning their time distance to a seizure event. The training phase used a hold-out or cross-fold validation method to get the required configuration parameters. Then, the classifier output was filtered and thresholded, to prevent rapid changes and thus, be robust to noise.

We replaced the filtering/decomposition, and feature extraction processes of the advisory system by our extracted features. By this way, we used the same data as in the EA. Then, from all possible combinations of features and electrodes, we selected the best set. Feature selection was based in two phases: using two filter methods[17] and an embedded one. Concerning the filter methods: firstly, we selected the best 100 features with Pearson's linear correlation coefficient and secondly, the best 25 with Area Under the Curve (AUC). Then, we used the wrapper method Recursive Feature Elimination (RFE) to select an optimal set of features. The filter method had the objective of substantially decreasing the number of features, and thus computational power, as it would take the RFE a substantially long period to compute. There are two important configuration options when using the RFE: the used algorithm to help selecting the features (the estimator), and the number of features. For the estimator, we used a linear Support Vector Machine (SVM)[1]. For the number of features, we used the first three chronological seizures for a grid-search (3, 5, 7, 10, 15, 20 features) that optimises the Sample Sensitivity (S_{ss}) and Sample Specificity (S_{sp}): $\sqrt{S_{ss} * S_{sp}}$ (also named here as fitness). We also used this grid search to find the best pre-ictal period (30, 35, 40, 45, 50, 55, 60 minutes). For the grid search, we used a 3-fold as in Direito et al.[1]: fold 1 used seizures #1 and #2 for training, and #3 for validation; fold 2 used seizures #1 and #3 for training, and #2 for validation; and fold 3 used seizures #2 and #3 for training, and #1 for validation. The remaining seizures comprised the testing group. Please note that we used the RFE with the SVM estimador instead of a backward feature elimination based on the BAHSIC criterion, as its code implementation was more easily available on Python scikit-learn library.

We adapted our classification model by using a Random Forest, since the one from the advisory system consists of an ensemble of a large number of individual decision tree classifiers. Each decision tree is trained with a different data distribution. Thus, we intended to mimic the ten layer structure along with the different decision surfaces, by using the random forest algorithm. In testing, we also implemented the Firing Power to smooth the output over time, with a similar threshold value to our approach (0.7). We also used refractory periods. This methodology is patient-specific.

Results

	Training			Testing		Control Method		
Patient ID	Pre-ictal (min)	Fitness (SS/SP/Comfort)	# Pareto Front Ind.	SS (0-1) FPR/h	Surrogate SS (0-1)	SS (0-1) FPR/h	Surrogate SS (0-1)	Above chance
**102**	**48.44±9.32**	**0.96±0.05**<br>**0.94±0.06**<br>**60.60±0.18**	**1671**	**0.21±0.12**<br>**0.24±0.12**	**0.19±0.05**	**0.13**<br>**0.09**	**0.07±0.26**	**MOEA**
**202**	**51.41±7.14**	**0.98±0.03**<br>**0.98±0.03**<br>**0.73±0.16**	**2569**	**0.00±0.06**<br>**0.14±0.09**	**0.12±0.09**	**1.00**<br>**0.00**	**0.07±0.25**	**Control**
500	51.53±9.60	0.98±0.02<br>0.99±0.01<br>0.63±0.18	2354	0.12±0.15<br>0.18±0.06	0.15±0.06	0.50<br>0.28	0.38±0.48	-
1200	50.02±7.52	0.95±0.05<br>0.95±0.05<br>0.57±0.19	1092	0.08±0.26<br>0.24±0.22	0.16±0.15	0.00<br>0.33	0.20±0.40	-
1500	49.46±8.08	0.98±0.02<br>0.99±0.02<br>0.71±0.18	2503	0.03±0.18<br>0.35±0.32	0.22±0.13	0.00<br>0.00	0.00±0.00	-
**1600**	**50.0±7.63**	**0.96±0.04**<br>**0.97±0.03**<br>**0.67±0.19**	**1801**	**0.12±0.32**<br>**0.04±0.07**	**0.03±0.06**	**0.00**<br>**1.19**	**0.43±0.50**	**MOEA**
2100	47.13±8.26	0.95±0.05<br>0.95±0.06<br>0.70±0.18	1452	0.19±0.19<br>0.24±0.11	0.18±0.08	0.00<br>0.05	0.03±0.18	-
2300	50.65±7.2	0.96±0.04<br>0.96±0.02<br>0.74±0.13	1558	0.06±0.24<br>0.30±0.30	0.19±0.12	0.00<br>0.38	0.53±0.50	-
**3300**	**50.86±9.44**	**0.96±0.04**<br>**0.96±0.03**<br>**0.69±0.16**	**1908**	**0.23±0.42**<br>**0.26±0.17**	**0.20±0.12**	**1.00**<br>**0.00**	**0.17±0.37**	**MOEA**<br>**Control**
4500	50.30±9.63	0.97±0.04<br>0.96±0.04<br>0.75±0.14	1727	0.18±0.38<br>0.33±0.16	0.24±0.11	0.00<br>0.69	0.43±0.50	-
5800	45.84±6.52	0.97±0.04<br>0.97±0.03<br>0.72±0.15	2090	0.12±0.21<br>0.35±0.15	0.24±0.13	0.00<br>0.21	0.15±0.36	-
**6000**	**52.99±9.36**	**0.97±0.03**<br>**0.96±0.03**<br>**0.73±0.15**	**1900**	**0.13±0.34**<br>**0.22±0.11**	**0.19±0.10**	**1.00**<br>**0.00**	**0.23±0.42**	**Control**
7200	50.38±8.5	0.97±0.03<br>0.97±0.03<br>0.72±0.15	2207	0.04±0.21<br>0.54±0.39	0.31±0.20	0.00<br>0.00	0.00±0.00	-
**8100**	**47.04±7.48**	**0.93±0.06**<br>**0.90±0.06**<br>**0.68±0.16**	**937**	**0.18±0.27**<br>**0.18±0.06**	**0.14±0.06**	**0.00**<br>**0.06**	**0.02±0.13**	**MOEA**
**8902**	**51.17±9.08**	**0.95±0.05**<br>**0.95±0.06**<br>**0.77±0.11**	**1154**	**0.20±0.27**<br>**0.16±0.09**	**0.24±0.08**	**1.00**<br>**0.00**	**0.05±0.22**	**Control**
11002	52.94±10.65	0.98±0.04<br>0.98±0.03<br>0.69±0.19	2507	0.00±0.03<br>0.27±0.19	0.20±0.13	0.00<br>0.31	0.17±0.37	-

11502	48.17±9.45	0.92±0.06<br>0.89±0.03<br>0.71±0.14	930	0.06±0.06<br>0.14±0.04	0.15±0.04	0.00<br>1.20	0.31±0.46	-
**12702**	**53.8±7.61**	**0.99±0.02<br>0.99±0.02<br>0.86±0.15**	**2499**	**0.16±0.17<br>0.21±0.06**	**0.16±0.05**	**0.33<br>0.43**	**0.18±0.38**	**Control**
21602	49.91±9.55	0.97±0.03<br>0.97±0.03<br>0.70±0.16	1896	0.16±0.14<br>0.32±0.09	0.27±0.06	0.00<br>0.21	0.08±0.28	-
**21902**	**51.40±9.55**	**0.95±0.04<br>0.95±0.04<br>0.67±0.19**	**1457**	**0.00±0.07<br>0.18±0.12**	**0.13±0.08**	**1.00<br>0.00**	**0.20±0.40**	**Control**
**22602**	**51.74±7.82**	**0.98±0.02<br>0.96±0.03<br>0.75±0.14**	**2030**	**0.17±0.26<br>0.10±0.08**	**0.17±0.07**	**0.50<br>0.00**	**0.10±0.30**	**Control**
23902	50.33±10.39	0.97±0.03<br>0.96±0.03<br>0.67±0.17	2120	0.08±0.18<br>0.22±0.09	0.23±0.13	0.00<br>3.43	0.43±0.50	-
**26102**	**49.11±7.34**	**0.91±0.06<br>0.91±0.05<br>0.63±0.15**	**684**	**0.13±0.33<br>0.34±0.11**	**0.24±0.10**	**1.00<br>0.00**	**0.17±0.37**	**Control**
**30802**	**51.73±8.31**	**0.98±0.03<br>0.97±0.05<br>0.64±0.17**	**1919**	**0.19±0.22<br>0.19±0.07**	**0.20±0.06**	**0.60<br>0.32**	**0.34±0.48**	**Control**
**32502**	**53.14±8.70**	**0.98±0.03<br>0.98±0.02<br>0.76±0.14**	**2570**	**0.12±0.18<br>0.35±0.13**	**0.27±0.08**	**0.33<br>0.91**	**0.17±0.37**	**Control**
**46702**	**51.05±8.08**	**0.97±0.03<br>0.96±0.03<br>0.72±0.17**	**1794**	**0.25±0.31<br>0.28±0.19**	**0.19±0.12**	**0.50<br>0.67**	**0.45±0.50**	**MOEA**
**50802**	**50.36±7.44**	**0.98±0.03<br>0.98±0.02<br>0.72±0.17**	**2221**	**0.03±0.13<br>0.22±0.09**	**0.15±0.07**	**0.50<br>1.45**	**0.20±0.40**	**Control**
51002	51.75±7.26	0.92±0.07<br>0.92±0.07<br>0.76±0.13	575	0.08±0.10<br>0.18±0.09	0.16±0.11	0.60<br>1.84	0.68±0.47	-
52302	51.0±8.17	0.98±0.03<br>0.98±0.02<br>0.75±0.14	2499	0.01±0.08<br>0.24±0.18	0.16±0.10	0.00<br>0.00	0.00±0.00	-
**53402**	**53.73±7.61**	**0.98±0.03<br>0.97±0.03<br>0.71±0.14**	**2447**	**0.30±0.33<br>0.26±0.06**	**0.20±0.06**	**0.50<br>0.03**	**0.02±0.13**	**MOEA<br>Control**
55202	53.26±9.34	0.95±0.05<br>0.95±0.04<br>0.69±0.17	1352	0.18±0.16<br>0.23±0.06	0.21±0.05	0.00<br>0.08	0.12±0.32	-
**56402**	**53.23±6.88**	**0.98±0.03<br>0.96±0.04<br>0.75±0.16**	**2105**	**0.05±0.21<br>0.14±0.10**	**0.12±0.10**	**1.00<br>0.36**	**0.27±0.44**	**Control**
58602	52.35±11.14	0.97±0.03<br>0.97±0.03<br>0.72±0.15	1369	0.05±0.12<br>0.25±0.10	0.23±0.11	0.00<br>0.49	0.21±0.41	-
63502	48.15±9.05	0.98±0.03<br>0.97±0.03<br>0.64±0.18	2374	0.10±0.21<br>0.14±0.06	0.13±0.07	0.50<br>2.43	0.48±0.50	-

64702	54.85±7.57	0.97±0.03<br>0.96±0.03<br>0.70±0.18	2008	0.08±0.19<br>0.14±0.07	0.13±0.08	0.50<br>0.76	0.38±0.49	-
**70302**	**46.97±8.51**	**0.96±0.04<br>0.93±0.06<br>0.64±0.15**	**570**	**0.13±0.10<br>0.26±0.06**	**0.18±0.06**	**0.43<br>0.25**	**0.19±0.39**	**Control**
**70902**	**48.34±7.79**	**0.98±0.03<br>0.98±0.02<br>0.71±0.20**	**1930**	**0.07±0.17<br>0.21±0.10**	**0.13±0.07**	**0.50<br>0.88**	**0.28±0.45**	**Control**
**71102**	**51.83±6.08**	**0.97±0.03<br>0.97±0.03<br>0.74±0.19**	**2090**	**0.11±0.21<br>0.19±0.12**	**0.18±0.08**	**1.00<br>1.22**	**0.62±0.49**	**Control**
71502	47.62±7.58	0.95±0.05<br>0.93±0.06<br>0.62±0.16	1451	0.05±0.14<br>0.16±0.07	0.14±0.06	0.50<br>10.14	0.33±0.47	-
**71802**	**51.71±8.48**	**0.95±0.04<br>0.96±0.04<br>0.70±0.20**	**1164**	**0.12±0.21<br>0.21±0.08**	**0.17±0.07**	**0.50<br>10.63**	**0.30±0.46**	**Control**
**73002**	**51.07±9.61**	**0.98±0.02<br>0.99±0.02<br>0.65±0.18**	**2560**	**0.04±0.14<br>0.17±0.08**	**0.14±0.08**	**1.00<br>1.30**	**0.20±0.40**	**Control**
**75102**	**50.25±8.93**	**0.97±0.03<br>0.97±0.03<br>0.69±0.18**	**1924**	**0.19±0.26<br>0.24±0.09**	**0.20±0.08**	**0.50<br>0.66**	**0.23±0.42**	**Control**
**75202**	**49.45±8.64**	**0.98±0.02<br>0.98±0.02<br>0.65±0.18**	**2225**	**0.19±0.17<br>0.14±0.05**	**0.15±0.06**	**0.00<br>0.17**	**0.07±0.25**	**MOEA**
**79502**	**47.62±10.08**	**0.94±0.06<br>0.87±0.06<br>0.69±0.17**	**1231**	**0.21±0.17<br>0.18±0.08**	**0.15±0.07**	**0.25<br>0.87**	**0.22±0.41**	**MOEA**
80602	52.12±9.73	0.98±0.02<br>0.99±0.02<br>0.76±0.15	2230	0.04±0.13<br>0.12±0.04	0.10±0.05	0.00<br>0.14	0.03±0.18	-
80702	50.65±8.26	0.96±0.04<br>0.95±0.05<br>0.73±0.18	1580	0.10±0.20<br>0.14±0.08	0.15±0.07	0.00<br>0.00	0.00±0.00	-
**81402**	**50.58±8.10**	**0.98±0.02<br>0.99±0.02<br>0.80±0.11**	**2110**	**0.41±0.26<br>0.19±0.05**	**0.16±0.05**	**0.33<br>0.00**	**0.08±0.27**	**MOEA<br>Control**
**85202**	**52.02±11.45**	**0.96±0.04<br>0.96±0.04<br>0.67±0.18**	**1389**	**0.42±0.40<br>0.25±0.13**	**0.21±0.09**	**0.00<br>3.82**	**0.32±0.46**	**MOEA**
**92102**	**48.98±7.10**	**0.97±0.03<br>0.96±0.03<br>0.70±0.16**	**1595**	**0.40±0.32<br>0.27±0.07**	**0.18±0.07**	**0.00<br>2.37**	**0.37±0.48**	**MOEA**
93402	50.31±8.29	0.99±0.02<br>0.99±0.02<br>0.82±0.10	2524	0.11±0.21<br>0.32±0.08	0.25±0.10	0.00<br>0.00	0.00±0.00	-
**93902**	**48.66±7.89**	**0.97±0.03<br>0.95±0.04<br>0.66±0.20**	**1852**	**0.37±0.28<br>0.23±0.15**	**0.20±0.10**	**0.00<br>2.28**	**0.47±0.50**	**MOEA**
94402	48.76±9.0	0.98±0.03<br>0.96±0.03<br>0.74±0.15	1748	0.13±0.16<br>0.36±0.13	0.24±0.07	0.00<br>1.42	0.48±0.50	-

95202	50.41±10.48	0.96±0.03<br>0.95±0.03<br>0.66±0.17	1144	0.09±0.14<br>0.16±0.06	0.13±0.06	0.00<br>0.02	0.00±0.00	-
96002	50.72±7.60	0.98±0.02<br>0.99±0.02<br>0.81±0.16	2725	0.16±0.18<br>0.22±0.06	0.16±0.05	0.00<br>0.30	0.14±0.35	-
**98102**	**51.01±8.16**	**0.97±0.04<br>0.93±0.05<br>0.65±0.19**	**1194**	**0.32±0.28<br>0.11±0.07**	**0.11±0.06**	**0.50<br>2.46**	**0.22±0.41**	**MOEA<br>Control**
100002	51.65±9.36	0.98±0.03<br>0.98±0.03<br>0.73±0.13	2472	0.13±0.10<br>0.27±0.06	0.18±0.05	0.00<br>0.15	0.10±0.30	-
**101702**	**49.54±8.12**	**0.96±0.04<br>0.96±0.04<br>0.64±0.18**	**1448**	**0.34±0.31<br>0.24±0.14**	**0.20±0.10**	**0.00<br>0.15**	**0.10±0.30**	**MOEA**
**102202**	**50.09±8.40**	**0.96±0.05<br>0.94±0.05<br>0.68±0.15**	**1544**	**0.22±0.22<br>0.18±0.06**	**0.15±0.06**	**0.00<br>0.06**	**0.03±0.16**	**MOEA**
**103002**	**54.48±9.39**	**0.98±0.03<br>0.98±0.02<br>0.70±0.14**	**2384**	**0.17±0.22<br>0.13±0.04**	**0.12±0.05**	**0.67<br>1.20**	**0.50±0.50**	**MOEA**
**103802**	**50.36±8.95**	**0.97±0.03<br>0.97±0.03<br>0.76±0.15**	**2084**	**0.26±0.24<br>0.18±0.11**	**0.16±0.07**	**0.00<br>0.06**	**0.00±0.00**	**MOEA**
**104602**	**52.63±8.68**	**0.97±0.04<br>0.98±0.03<br>0.74±0.16**	**2071**	**0.33±0.29<br>0.26±0.16**	**0.23±0.09**	**0.50<br>0.00**	**0.10±0.30**	**MOEA<br>Control**
109202	52.89±9.84	0.95±0.03<br>0.95±0.04<br>0.72±0.14	1391	0.06±0.24<br>0.34±0.19	0.25±0.15	0.00<br>0.00	0.00±0.00	-
109502	54.82±8.58	0.98±0.02<br>0.99±0.02<br>0.80±0.14	2405	0.11±0.31<br>0.14±0.08	0.13±0.08	0.00<br>0.30	0.03±0.18	-
**109602**	**53.16±7.93**	**0.99±0.01<br>0.99±0.01<br>0.86±0.11**	**2373**	**0.26±0.44<br>0.16±0.06**	**0.14±0.08**	**1.00<br>1.54**	**0.47±0.50**	**MOEA<br>Control**
**110602**	**52.32±8.01**	**0.98±0.03<br>0.98±0.03<br>0.74±0.14**	**2256**	**0.37±0.29<br>0.20±0.08**	**0.19±0.07**	**0.00<br>0.00**	**0.47±0.00**	**MOEA**
**111902**	**49.25±9.42**	**0.95±0.06<br>0.93±0.06<br>0.59±0.19**	**1361**	**0.08±0.19<br>0.12±0.13**	**0.13±0.11**	**0.50<br>0.00**	**0.07±0.25**	**Control**
112402	51.96±9.37	0.96±0.05<br>0.96±0.05<br>0.68±0.13	1168	0.16±0.12<br>0.25±0.09	0.19±0.06	0.22<br>0.32	0.20±0.40	-
**113902**	**52.06±7.22**	**0.97±0.03<br>0.97±0.03<br>0.74±0.13**	**2229**	**0.28±0.12<br>0.07±0.06**	**0.07±0.05**	**0.00<br>0.00**	**0.00±0.00**	**MOEA**
**114702**	**47.77±7.98**	**0.98±0.02<br>0.98±0.03<br>0.76±0.21**	**2541**	**0.16±0.16<br>0.35±0.12**	**0.24±0.08**	**0.40<br>0.60**	**0.17±0.38**	**Control**
**114902**	**52.95±8.07**	**0.98±0.03<br>0.98±0.03<br>0.77±0.15**	**2566**	**0.33±0.21<br>0.10±0.04**	**0.09±0.04**	**0.25<br>0.55**	**0.16±0.36**	**MOEA**

**115202**	**50.25±9.16**	**0.96±0.03**<br>**0.97±0.04**<br>**0.80±0.19**	**1610**	**0.25±0.18**<br>**0.22±0.08**	**0.19±0.06**	**0.00**<br>**0.17**	**0.06±0.23**	**MOEA**
123902	51.17±9.63	0.98±0.02<br>0.99±0.02<br>0.71±0.15	2521	0.07±0.18<br>0.14±0.08	0.11±0.08	0.50<br>0.25	0.33±0.47	-
125002	51.5±10.05	0.97±0.03<br>0.97±0.03<br>0.64±0.16	1622	0.14±0.15<br>0.20±0.09	0.17±0.07	0.25<br>1.80	0.27±0.44	-
1235103	51.25±8.43	0.98±0.03<br>0.98±0.02<br>0.71±0.16	2226	0.09±0.22<br>0.13±0.08	0.11±0.08	0.50<br>7.91	0.75±0.43	-
1307103	51.08±9.03	0.97±0.05<br>0.95±0.06<br>0.71±0.14	1998	0.10±0.12<br>0.11±0.06	0.16±0.04	0.20<br>0.13	0.33±0.47	-
**1308503**	**52.06±9.81**	**0.98±0.02**<br>**0.99±0.02**<br>**0.75±0.12**	**2619**	**0.26±0.44**<br>**0.20±0.13**	**0.15±0.11**	**1.00**<br>**2.39**	**0.43±0.50**	**MOEA**<br>**Control**
**1308603**	**50.32±8.75**	**0.98±0.03**<br>**0.98±0.03**<br>**0.73±0.17**	**2093**	**0.41±0.49**<br>**0.19±0.09**	**0.15±0.08**	**0.00**<br>**0.36**	**0.20±0.40**	**MOEA**
**1310803**	**50.51±7.93**	**0.95±0.06**<br>**0.94±0.06**<br>**0.61±0.15**	**1770**	**0.01±0.06**<br>**0.20±0.07**	**0.17±0.10**	**1.00**<br>**0.00**	**0.10±0.30**	**Control**
**1312903**	**50.31±7.82**	**0.98±0.02**<br>**0.98±0.02**<br>**0.66±0.18**	**2363**	**0.12±0.32**<br>**0.12±0.09**	**0.09±0.08**	**0.00**<br>**0.33**	**0.37±0.48**	**MOEA**
1315203	53.08±7.29	0.98±0.03<br>0.97±0.04<br>0.67±0.17	2072	0.06±0.24<br>0.13±0.07	0.11±0.08	0.00<br>0.00	0.00±0.00	-
**1319203**	**50.42±6.78**	**0.95±0.05**<br>**0.95±0.05**<br>**0.64±0.20**	**1385**	**0.01±0.06**<br>**0.20±0.07**	**0.09±0.08**	**0.50**<br>**0.23**	**0.28±0.45**	**Control**
**1321803**	**49.79±8.43**	**0.97±0.03**<br>**0.97±0.03**<br>**0.67±0.19**	**2522**	**0.54±0.50**<br>**0.14±0.26**	**0.18±0.14**	**1.00**<br>**0.38**	**0.23±0.42**	**MOEA**<br>**Control**
**1322803**	**51.59±8.8**	**0.98±0.03**<br>**0.97±0.03**<br>**0.74±0.17**	**2471**	**0.09±0.28**<br>**0.24±0.12**	**0.19±0.11**	**1.00**<br>**0.00**	**0.10±0.30**	**Control**
1324903	50.95±8.00	0.91±0.06<br>0.95±0.05<br>0.66±0.15	880	0.25±0.30<br>0.22±0.10	0.23±0.08	0.00<br>1.63	0.28±0.45	-
1325103	51.53±8.27	0.96±0.04<br>0.92±0.05<br>0.70±0.19	1275	0.09±0.22<br>0.13±0.07	0.11±0.08	0.00<br>0.30	0.10±0.30	-
**1325403**	**48.62±8.17**	**0.97±0.03**<br>**0.98±0.02**<br>**0.73±0.17**	**2197**	**0.21±0.41**<br>**0.28±0.29**	**0.18±0.16**	**0.00**<br>**0.00**	**0.00±0.00**	**MOEA**
**1325603**	**48.41±8.99**	**0.94±0.05**<br>**0.95±0.05**<br>**0.66±0.21**	**1190**	**0.12±0.32**<br>**0.28±0.29**	**0.20±0.22**	**1.00**<br>**40.00**	**0.60±0.49**	**Control**
1326103	49.25±8.70	0.97±0.03<br>0.96±0.03<br>0.77±0.16	1510	0.09±0.28<br>0.30±0.13	0.21±0.10	0.00<br>0.00	0.00±0.00	-

1327403	50.25±9.42	0.95±0.05<br>0.95±0.04<br>0.62±0.16	1001	0.00±0.00<br>0.10±0.10	0.09±0.10	0.00<br>0.34	0.20±0.40	-
**1328603**	**50.18±9.70**	**0.95±0.06**<br>**0.93±0.06**<br>**0.67±0.16**	**1334**	**0.16±0.37**<br>**0.33±0.13**	**0.24±0.11**	**1.00**<br>**1.90**	**0.63±0.48**	**Control**
1328803	52.76±8.97	0.99±0.02<br>0.99±0.02<br>0.79±0.12	2658	0.04±0.20<br>0.20±0.24	0.13±0.15	0.00<br>0.69	0.10±0.30	-
**1328903**	**50.15±9.77**	**0.97±0.03**<br>**0.98±0.02**<br>**0.71±0.20**	**2304**	**0.23±0.28**<br>**0.20±0.05**	**0.15±0.07**	**0.00**<br>**1.21**	**0.23±0.42**	**MOEA**
**1330903**	**46.88±8.63**	**0.98±0.03**<br>**0.98±0.03**<br>**0.67±0.19**	**2076**	**0.19±0.15**<br>**0.13±0.04**	**0.08±0.03**	**0.20**<br>**0.07**	**0.08±0.27**	**MOEA**<br>**Control**

Table S 2. The results for all patients.

Phenotype study for all patients

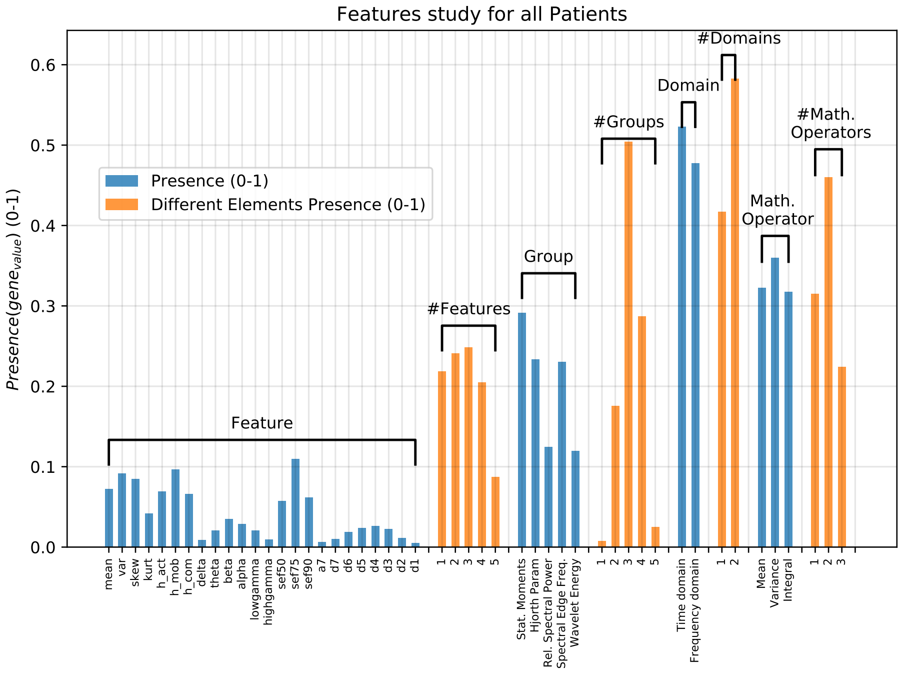

S 1. Phenotype feature study for all patients. The presence of the gene values are presented in blue. The simultaneous presence of different gene values is presented in orange.

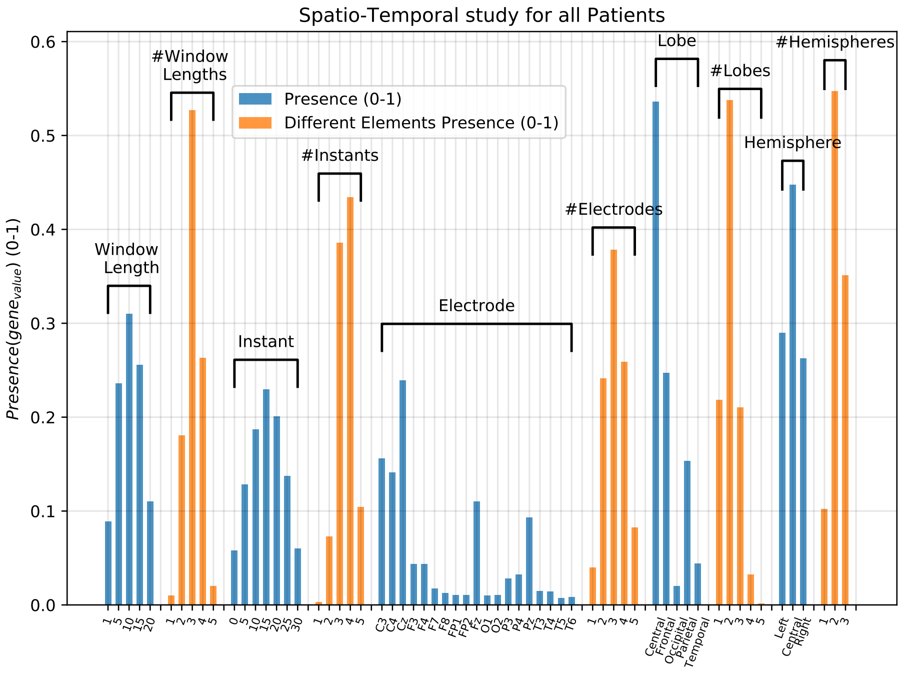

S 2. Phenotype temporal and spatial study for all patients. The presence of temporal (window length and time instant) and spatial (electrode, lobe, and hemisphere) gene values are presented in blue. The simultaneous presence of different gene values is presented in orange.

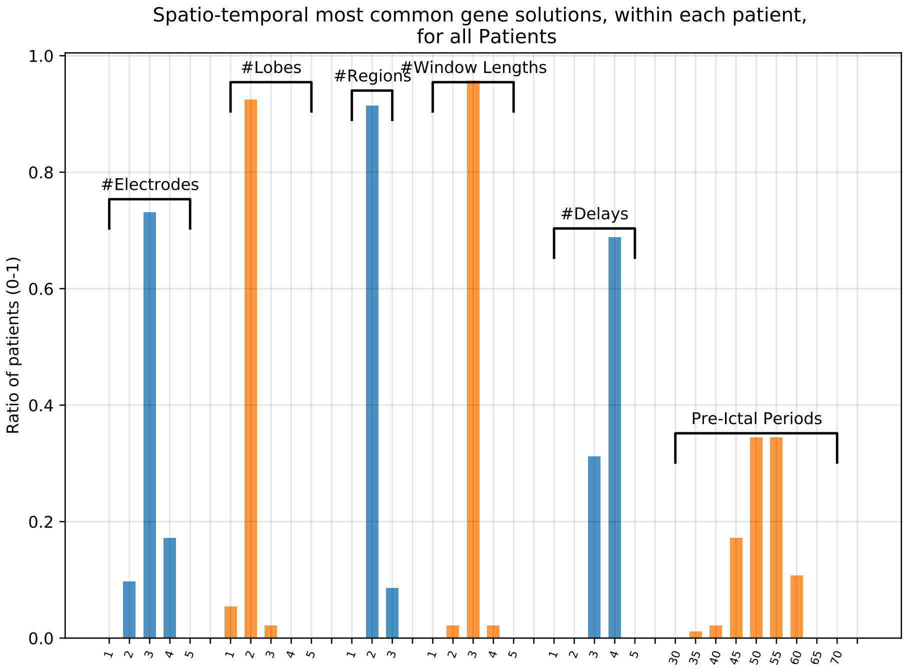

S 3. The most common gene solutions, for each patient, for all patients concerning the number of simultaneously used different electrodes, lobes, regions, window lengths, and delays. The most common pre-ictal period, for each patient, is also presented.

Pre-Ictal Period Discussion

We present here some examples of the pre-ictal periods' histograms for all patients, specifically for patients 21602, 21902, 30802, and 32502, which had, as mean pre-ictal period the following values, respectively: 49.91±9.55, 51.40±9.55, 51.73±8.31, and 53.14±8.70 minutes. In the GitHub page of our paper, you can find these histograms for all patients. We also present the most frequent pre-ictal period (the mode) for each patient. The MOEA can find solutions for many possible pre-ictal periods in all patients.

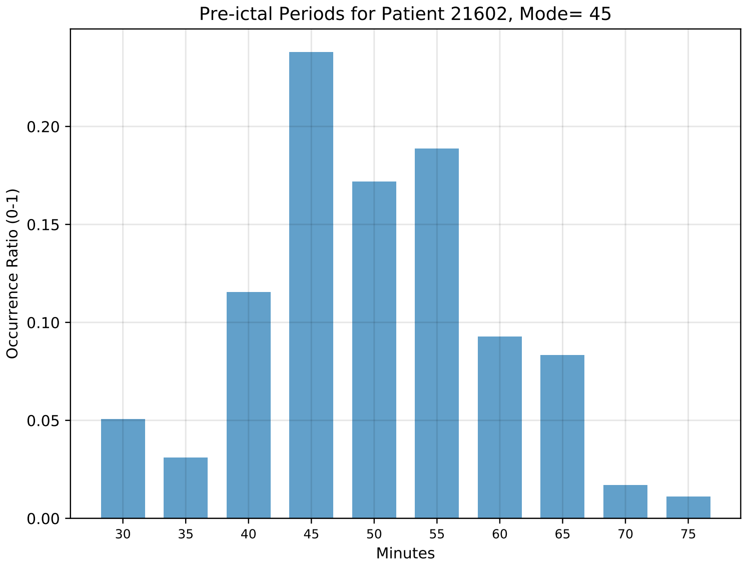

S 4. The obtained pre-ictal periods, for all MOEA solutions, for patient 21602.

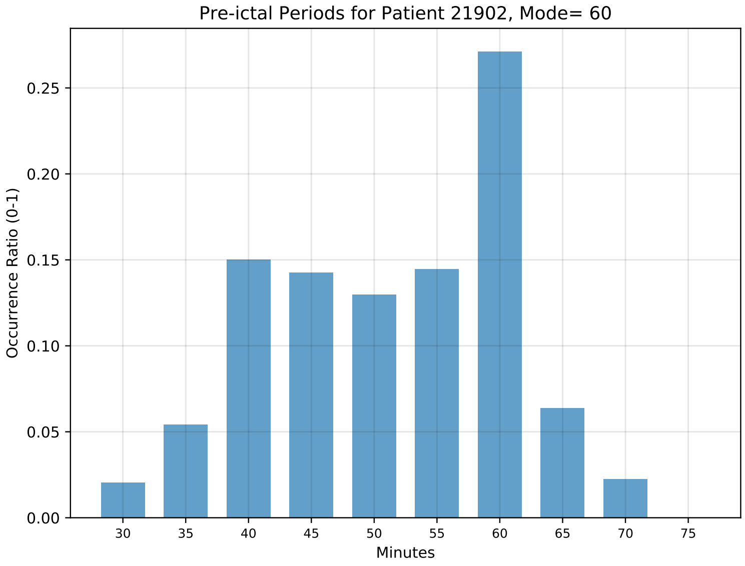

S 5. The obtained pre-ictal periods, for all MOEA solutions, for patient 21902.

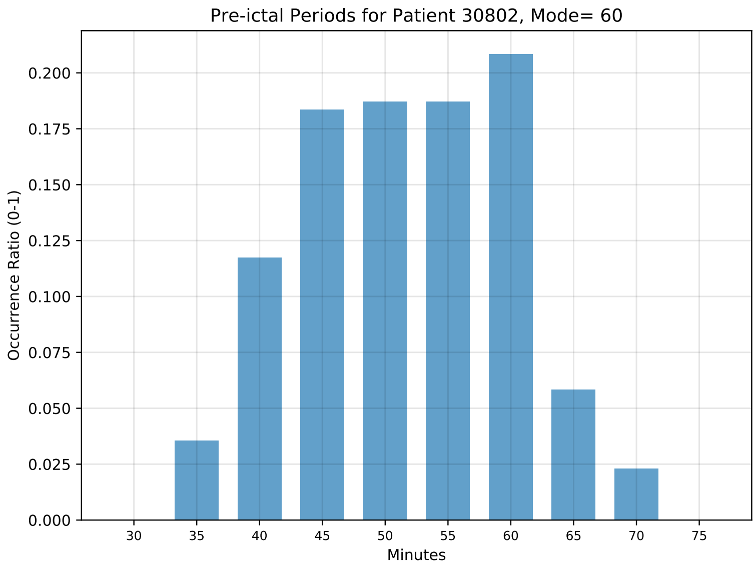

S 6. The obtained pre-ictal periods, for all MOEA solutions, for patient 30802.

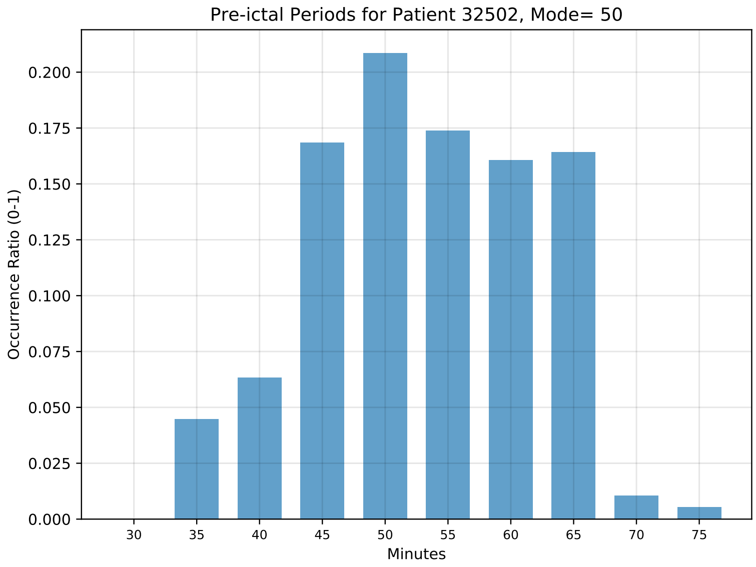

S 7. The obtained pre-ictal periods, for all MOEA solutions, for patient 32502.

Electrodes and Lobes Discussion

We present here some examples of the number of different electrodes and lobes histograms for all patients, specifically for patients 1200, 12702, 55202, 81402, and 1319203, which had, as most common set (mode) of electrodes and lobes the following values, respectively: 4 and 3; 2 and 1; 3 and 2; 2 and 2; and 4 and 2. On the GitHub page of our paper, one can find these histograms for all patients. We believe these findings evidence the relevance of the patient comfort metric in the MOEA.

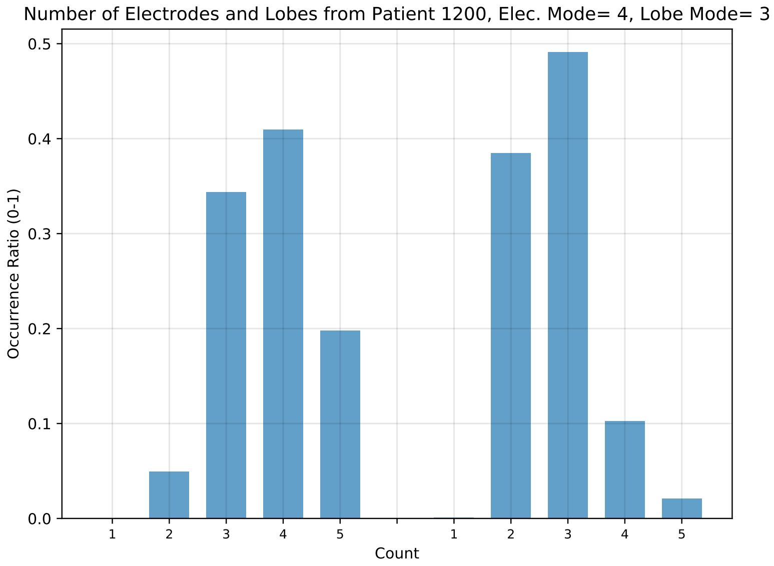

S 8. The obtained electrode and lobe study, for all MOEA solutions, for patient 1200. On the left, the number of different electrodes. On the right, the number of different lobes.

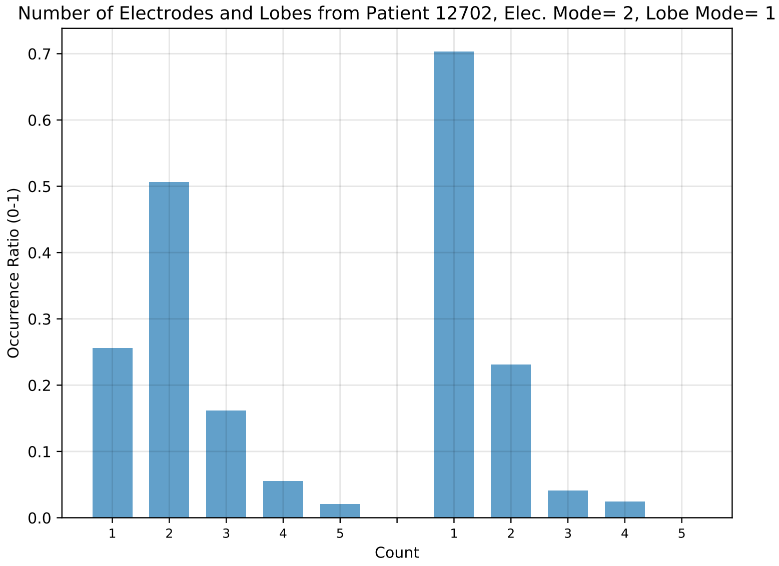

S 9. The obtained electrode and lobe study, for all MOEA solutions, for patient 12702. On the left, the number of different electrodes. On the right, the number of different lobes.

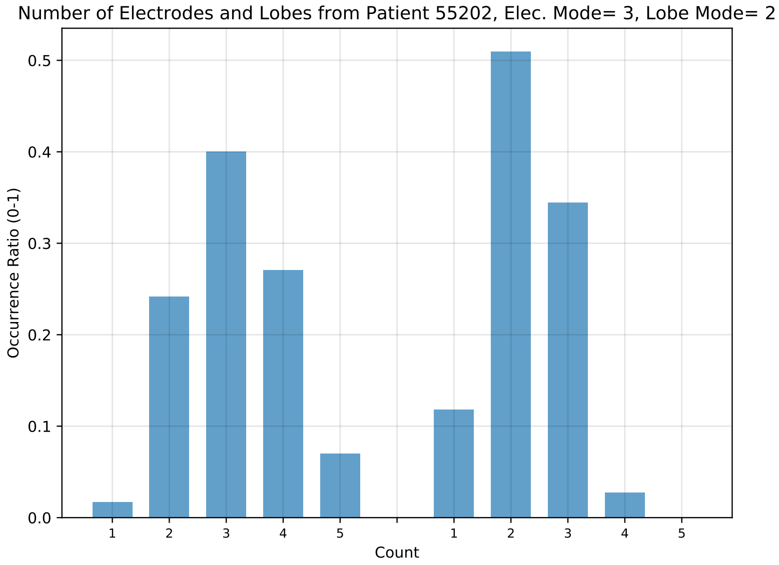

S 10. The obtained electrode and lobe study, for all MOEA solutions, for patient 55202. On the left, the number of different electrodes. On the right, the number of different lobes.

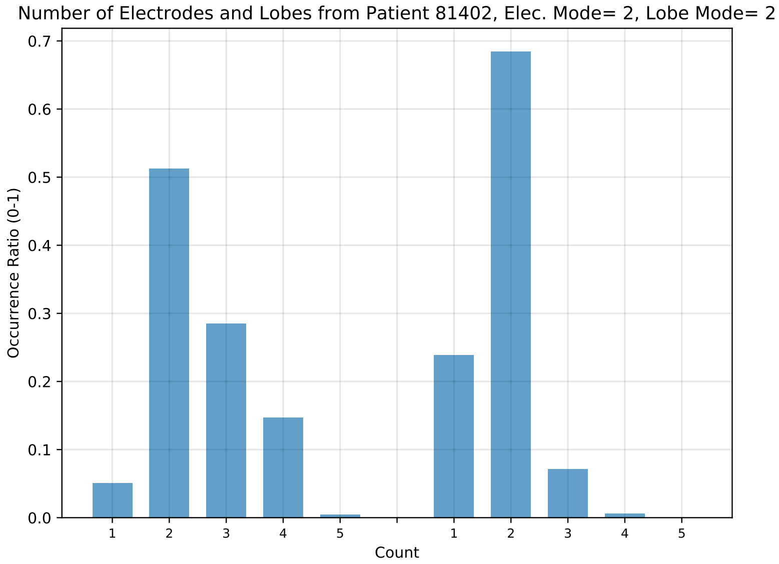

S 11. The obtained electrode and lobe study, for all MOEA solutions, for patient 81402. On the left, the number of different electrodes. On the right, the number of different lobes.

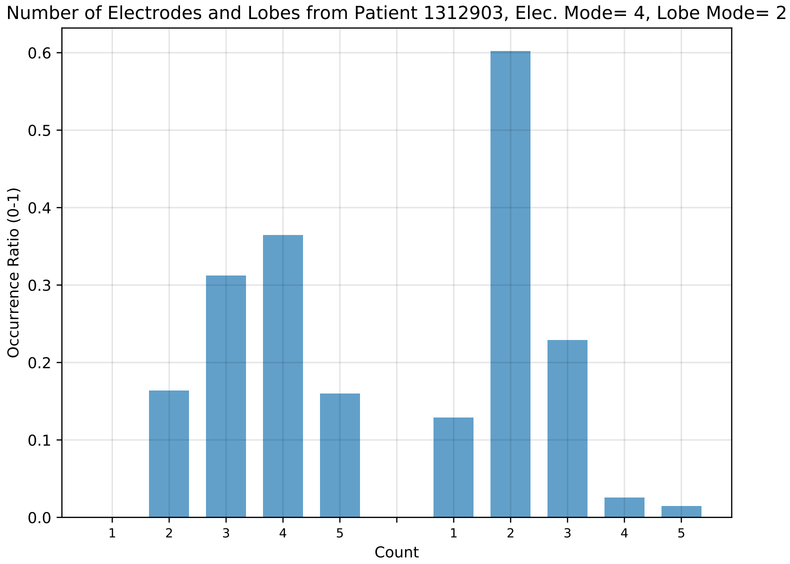

S 12. The obtained electrode and lobe study, for all MOEA solutions, for patient 1312903. On the left, the number of different electrodes. On the right, the number of different lobes.

Impact of Comfort in Performance

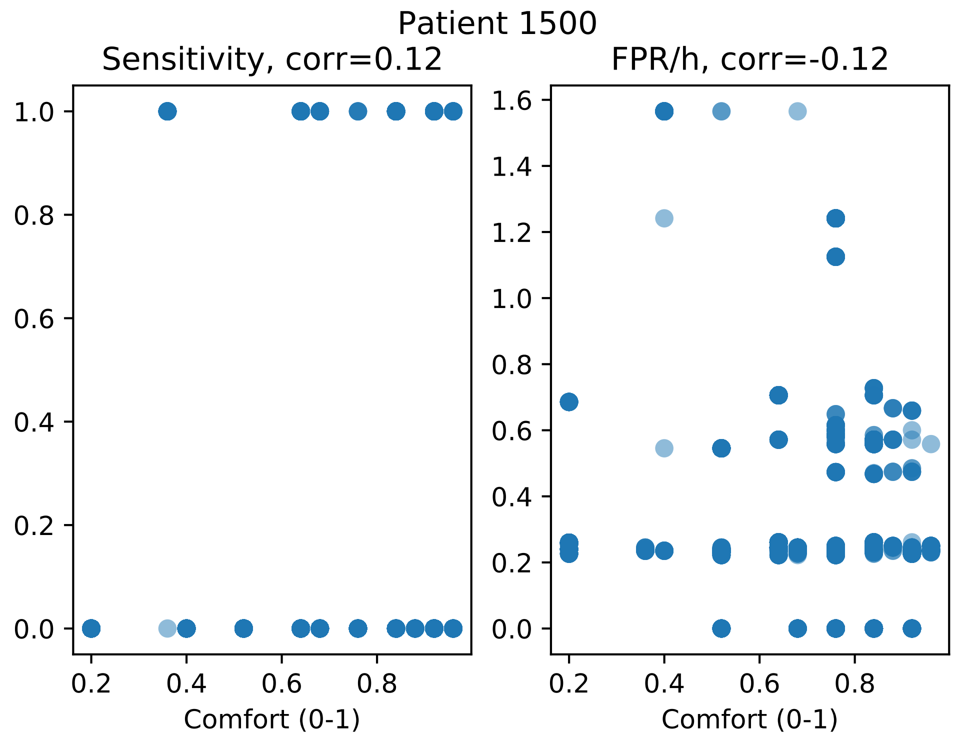

S 13. The impact of comfort in performance for patient 1500. The overall performance increased.

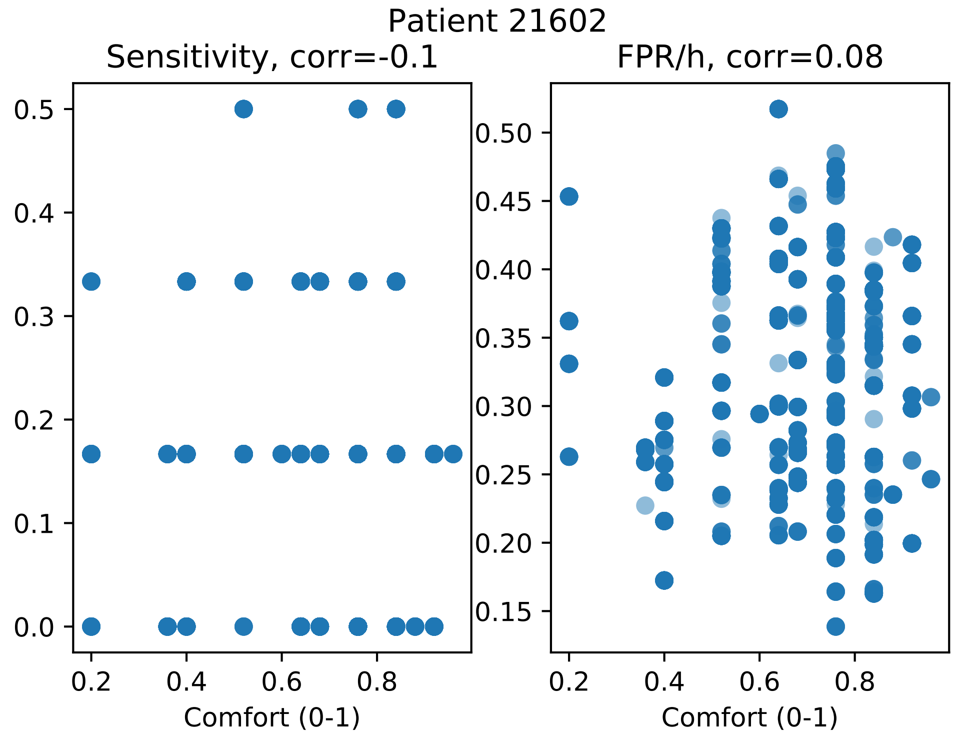

S 14. The impact of comfort in performance for patient 21602. The overall performance was maintained.

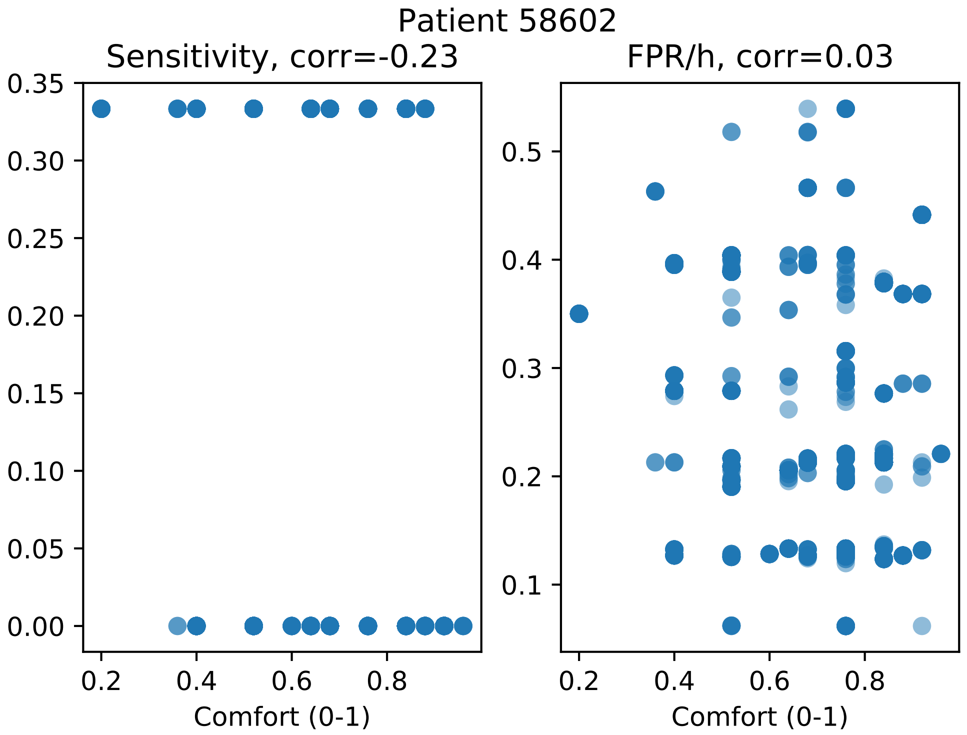

S 15. The impact of comfort in performance for patient 58602. The overall performance decreased.

References

1. Direito, B., Teixeira, C. A., Sales, F., Castelo-Branco, M. & Dourado, A. A Realistic Seizure Prediction Study Based on Multiclass SVM. *Int. J. Neural Syst.* **27**, 1750006, DOI: 10.1142/S012906571750006X (2017).
2. Mormann, F., Andrzejak, R. G., Elger, C. E. & Lehnertz, K. Seizure Prediction: The Long and Winding Road. *Brain* **130**, 314–333, DOI: 10.1093/brain/awl241 (2007).
3. Rasekhi, J., Mollaei, M. R. K., Bandarabadi, M., Teixeira, C. A. & Dourado, A. Preprocessing Effects of 22 Linear Univariate Features on the Performance of Seizure Prediction Methods. *J. Neurosci. Methods* **217**, 9–16, DOI: 10.1016/j.jneumeth.2013.03.019 (2013).
4. Teixeira, C. *et al.* Epileptic Seizure Predictors Based on Computational Intelligence Techniques: A Comparative Study with 278 Patients. *Comput. Methods Programs Biomed.* **114**, 324–336, DOI: 10.1016/j.cmpb.2014.02.007 (2014).
5. Mormann, F. *et al.* On the predictability of epileptic seizures. *Clin. neurophysiology* **116**, 569–587 (2005).
6. Moghim, N. & Corne, D. W. Predicting epileptic seizures in advance. *PloS one* **9**, e99334 (2014).
7. Bou Assi, E., Nguyen, D. K., Rihana, S. & Sawan, M. Towards Accurate Prediction of Epileptic Seizures: A Review. *Biomed. Signal Process. Control.* **34**, 144–157, DOI: 10.1016/j.bspc.2017.02.001 (2017).
8. Gadhoumi, K., Lina, J. M., Mormann, F. & Gotman, J. Seizure Prediction for Therapeutic Devices: A Review. *J. Neurosci. Methods* **260**, 270–282, DOI: 10.1016/j.jneumeth.2015.06.010 (2016).
9. Aarabi, A., Fazel-Rezai, R. & Aghakhani, Y. Eeg seizure prediction: measures and challenges. In *2009 Annual International Conference of the IEEE Engineering in Medicine and Biology Society*, 1864–1867 (IEEE, 2009).
10. Bandarabadi, M., Teixeira, C. A., Rasekhi, J. & Dourado, A. Epileptic seizure prediction using relative spectral power features. *Clin. Neurophysiol.* **126**, 237–248 (2015).
11. Howbert, J. J. *et al.* Forecasting seizures in dogs with naturally occurring epilepsy. *PloS one* **9**, e81920 (2014).
12. Park, Y., Luo, L., Parhi, K. K. & Netoff, T. Seizure prediction with spectral power of eeg using cost-sensitive support vector machines. *Epilepsia* **52**, 1761–1770 (2011).
13. Gadhoumi, K., Lina, J.-M. & Gotman, J. Seizure prediction in patients with mesial temporal lobe epilepsy using eeg measures of state similarity. *Clin. Neurophysiol.* **124**, 1745–1754 (2013).
14. Deb, K., Pratap, A., Agarwal, S. & Meyarivan, T. A fast and elitist multiobjective genetic algorithm: Nsga-ii. *IEEE transactions on evolutionary computation* **6**, 182–197 (2002).
15. Pinto, M. F. *et al.* A personalized and evolutionary algorithm for interpretable eeg epilepsy seizure prediction. *Sci. reports* **11**, 1–12 (2021).
16. Cook, M. J. *et al.* Prediction of Seizure Likelihood with a Long-Term, Implanted Seizure Advisory System in Patients with Drug-Resistant Epilepsy: a First-in-Man Study. *The Lancet Neurol.* **12**, 563–571 (2013).
17. Dash, M. & Liu, H. Feature Selection for Classification. *Intell. data analysis* **1**, 131–156 (1997).
